# Prevention of age-associated neuronal hyperexcitability with improved learning and attention upon knockout or antagonism of LPAR2

**DOI:** 10.1007/s00018-020-03553-4

**Published:** 2020-05-28

**Authors:** Caroline Fischer, Heiko Endle, Lana Schumann, Annett Wilken-Schmitz, Julia Kaiser, Susanne Gerber, Christina F. Vogelaar, Mirko H. H. Schmidt, Robert Nitsch, Isabel Snodgrass, Dominique Thomas, Johannes Vogt, Irmgard Tegeder

**Affiliations:** 1 Institute of Clinical Pharmacology, Goethe-University Frankfurt, Faculty of Medicine, Frankfurt, Germany; 2 Institute for Microscopic Anatomy and Neurobiology, University Medical Center of the Johannes Gutenberg University, Mainz, Germany; 3 Institute for Human Genetics, University Medical Center, Johannes Gutenberg University, Mainz, Germany; 4 Department of Neurology, University Medical Center of the Johannes Gutenberg University, Mainz, Germany; 5 Institute of Anatomy, Medical Faculty Carl Gustav Carus Technische Universität, School of Medicine, Dresden, Germany; 6 Institute for Translational Neuroscience, Westfälische Wilhelms Universität, Münster, Germany; 7 Center of Anatomy, University of Cologne, Cologne, Germany

**Keywords:** Lysophosphatidic acids, Cognition, Touchscreen, IntelliCage, Long-term potentiation, Hippocampal excitability

## Abstract

Recent studies suggest that synaptic lysophosphatidic acids (LPAs) augment glutamate-dependent cortical excitability and sensory information processing in mice and humans via presynaptic LPAR2 activation. Here, we studied the consequences of LPAR2 deletion or antagonism on various aspects of cognition using a set of behavioral and electrophysiological analyses. Hippocampal neuronal network activity was decreased in middle-aged LPAR2^−/−^ mice, whereas hippocampal long-term potentiation (LTP) was increased suggesting cognitive advantages of LPAR2^−/−^ mice. In line with the lower excitability, RNAseq studies revealed reduced transcription of neuronal activity markers in the dentate gyrus of the hippocampus in naïve LPAR2^−/−^ mice, including ARC, FOS, FOSB, NR4A, NPAS4 and EGR2. LPAR2^−/−^ mice behaved similarly to wild-type controls in maze tests of spatial or social learning and memory but showed faster and accurate responses in a 5-choice serial reaction touchscreen task requiring high attention and fast spatial discrimination. In IntelliCage learning experiments, LPAR2^−/−^ were less active during daytime but normally active at night, and showed higher accuracy and attention to LED cues during active times. Overall, they maintained equal or superior licking success with fewer trials. Pharmacological block of the LPAR2 receptor recapitulated the LPAR2^−/−^ phenotype, which was characterized by economic corner usage, stronger daytime resting behavior and higher proportions of correct trials. We conclude that LPAR2 stabilizes neuronal network excitability upon aging and allows for more efficient use of resting periods, better memory consolidation and better  performance in tasks requiring high selective attention. Therapeutic LPAR2 antagonism may alleviate aging-associated cognitive dysfunctions.

**Electronic supplementary material:**

The online version of this article (10.1007/s00018-020-03553-4) contains supplementary material, which is available to authorized users.

## Introduction

Lysophosphatidic acids (LPAs) strengthen glutamatergic synaptic neurotransmission and plasticity in the cortex and hippocampus via a presynaptic process involving an LPA receptor-2 (LPAR2)-evoked enhancement of glutamate release [1–3]. Via this loop, LPAs modulate neuronal network excitability and memory formation [4] as well as cortical stimulus processing and filtering [1, 3], suggesting that LPAs influence thereby the capability of selective attention and learning. LPAs can be generated within the synaptic cleft from lysophosphatidylcholines (LPC) of different chain lengths and saturation, via the ectonucleotide pyrophosphatase-phosphodiesterase 2 (ENPP2) known as autotaxin, which was found in astrocytic processes ensheathing glutamatergic cortical and hippocampal synapses [5]. This localization is optimal for local regulation of excitatory cortical transmission owing to autotaxin's ability to attach to activated membranes via integrins near its release site [6] and to recruit and prefer specific LPC species [7, 8], which are actively imported through the blood–brain barrier [9]. Autotaxin is highly expressed at glutamatergic cortical layer IV synapses and in the hippocampus [5]. At these sites, synaptic LPAs stimulate the presynaptic glutamate release via activation of the presynaptic G-protein coupled, LPAR2 [10]. In turn, synaptic LPAs are under the control of a LPA interacting molecule, plasticity-related gene 1 (PRG1), which regulates synaptic LPA content and thereby presynaptic LPAR2 activity via cellular import [3, 10]. The above-described synaptic lipid-signaling loop was shown to regulate the cortical excitation–inhibition balance and sensory information processing [1, 3].

Deficiency of PRG1-dependent postsynaptic LPA import results in LPAR2-dependent cortical network hyperexcitability leading to increased neuronal network synchronization up to epileptic discharges [2, 10]. The translational relevance of this LPAR2-regulated glutamatergic transmission was revealed by a human loss-of-function mutation of PRG1 (PRG1-R345T) [3], which results in cortical hyperexcitability and impaired somatosensory filter functions, specifically pre-pulse inhibition, a classical test in rodent models of schizophrenia and in clinical diagnosis of schizophrenia [5]. Hence, synaptic signaling through autotaxin/PRG1/LPAR2 appears to act as a sensory gate, and is, therefore, a putative therapeutic target for states of cortical hyperexcitability and subsequent sensory overflow or attentional deficits. Interestingly, hyperexcitability of cortical or hippocampal neurons was described as a critical factor for psychiatric disorders [11] and is a classical feature of neurodegenerative diseases like Alzheimer’s dementia [12–17], but also occurs upon normal aging in rodents [18, 19] and primates [20].

To address long-term effects of cortical LPAR2-regulated glutamatergic transmission, we assessed the consequences of LPAR2 deletion in mice in terms of behavioral and electrophysiological correlates of multiple aspects of spatial, social and discriminative cognition using a set of standard and advanced behavioral analyses, and recordings of hippocampal long-term potentiation (LTP) and network excitability. The data reveal that loss of LPAR2 preserves "youthful" network excitability in aging mice and leads to higher LTP, and enhances daytime resting behavior with better performance in selective attentional tasks and faster learning during circadian active time. Pharmacologic inhibition of LPAR2 in old mice partly recapitulates the beneficial reposing and pro-learning effects, particularly under sleep disrupting mild stress.

## Results

### Inverse mutual relationship of hippocampal excitability and long-term potentiation

**Fig. 1:**
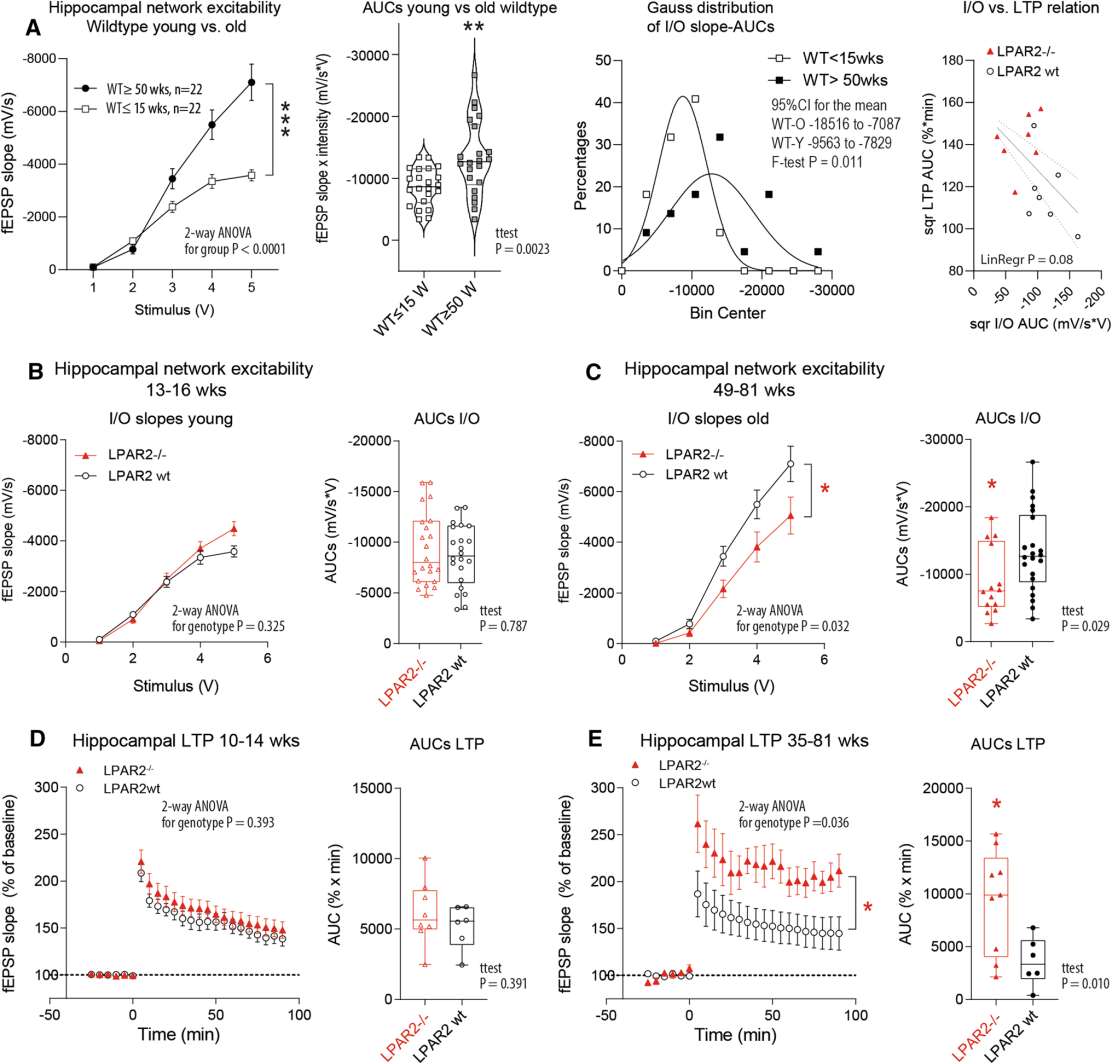
Field potentials in hippocampal brain slices. **a** Input versus output (*I/O*) curves of the initial slope of the field excitatory postsynaptic potential (fEPSP) in hippocampal brain slices of young versus middle-aged wild-type mice (mean ± SEM). The corresponding violin plots show the AUCs of individual slices. The line is the median. The gray lines show the interquartile range and scatter show individual slices. The frequency distribution of the AUCs was plotted according to Gauss. The Gauss distribution of middle-aged wild-type mice was significantly shifted to the right indicating higher excitability. Right: Relationship of *I/O* and LTP in wild-type (LPAR2 wt) and LPAR2^−/−^ slices. The data show the square roots (sqr) of the AUCs of *I/O* curves versus AUCs of LTP time courses. The lines show the linear regression line with 90% CI. **b**,** c** Input versus output (*I/O*) curves of the initial slope of the fEPSP in hippocampal brain slices of young and middle-aged wild-type (LPAR2 wt) and LPAR2^−/−^ mice (ages as indicated). The initial slopes of the fEPSP were expressed as percentages of the baseline average. Data are the mean and sem. The corresponding box/scatter plots show the AUCs of individual mice **d**,** e** Field excitatory postsynaptic potentials (fEPSPs, mean ± SEM) of hippocampal CA1 pyramidal neurons of wild-type (LPAR2 wt) and LPAR2^−/−^ hippocampal brain slices. Slices were prepared from young adult mice and from middle-aged to aged mice (ages as indicated). The box/scatter plots show the area under the curves from time zero to the end. Data were compared with two-way ANOVA followed by post hoc *t* tests using an adjustment of P according to Šidák. The linear trapezoidal rule was used for calculation of AUCs, which were compared with two-tailed unpaired *t* tests. Asterisks show significant differences with *P < 0.05, **P < 0.01, ***P < 0.001

In line with the data published by others [18, 21], we observed an age-dependent increase of hippocampal network excitability in wild-type animals (Fig. 1a). The *I/O* slopes differed significantly in slices of young WT versus middle-aged WT mice. Hyperexcitability in the CA3 region of the hippocampus and in the cortex has been associated with memory impairment [19, 21] and prodromal stages of neurodegenerative diseases [17, 22]. Motivated by the described role of LPAR2 in glutamatergic synapses, we assessed the excitability of young (≤ 15 weeks) and of middle-aged (≥ 50 weeks) LPAR2^−/−^ mice in comparison with the respective wild-type control mice (Fig. 1b, c). Excitability was similar in slices of young mice (Fig. 1b) but hippocampal network excitability was significantly lower in slices of middle-aged LPAR2^−/−^ mice (≥ 50 weeks) as compared with the respective wild-type controls (Fig. 1c). ANOVA results are shown in the figures.

A number of studies suggest that hippocampal neuron hyperexcitability impairs long-term potentiation (LTP) [23, 24], which is a neural correlate of synaptic plasticity underlying learning processes [25]. Indeed, regression analysis of excitability (*I/O*) versus LTP suggested an inverse relationship (Fig. 1a, right). LTP formation decreases upon aging [25, 26]. Therefore, we assessed LTP at CA3–CA1 synapses upon stimulation of Schaffer collaterals in slices from WT and LPAR2^−/−^ mice from different ages. Recordings of field excitatory postsynaptic potentials (fEPSP) were similar in slices of young adult mice (10–15 weeks), but comparisons of fEPSP in slices of mice beyond 35 weeks of age (35–81 weeks) revealed significantly stronger LTP in LPAR2^−/−^ slices as compared with wild-type slices (Fig. 1d, e). Hence, deletion of LPAR2 might prevent aging-associated raises of neuronal excitability, which is associated with higher LTP. The relative stronger hippocampal LTP in slices of LPAR2^−/−^ mice beyond 35 weeks of age suggested benefits in terms of learning and memory [25, 27–29].

Consequently, we assessed the behavior in middle-aged to old wild-type and LPAR2-deficient mice. Maze-based standard tests of spatial learning (Suppl. Figure 1A, B) or social learning (Suppl. Figure 1C) did not show significant differences, but a significant reduction of shelter seeking behavior in the zero-maze test (Suppl. Figure 1D) pointed to lower anxiety, which might affect the learning drive in the Barnes Maze. We, therefore, set out to investigate the behavior in more detail using IntelliCage and touchscreen tasks, in which appetitive drive, attention, social and circadian influences are monitored and balanced.

### Lower exploratory but stronger goal-directed activity: more efficient use of visits for licking

**Fig. 2:**
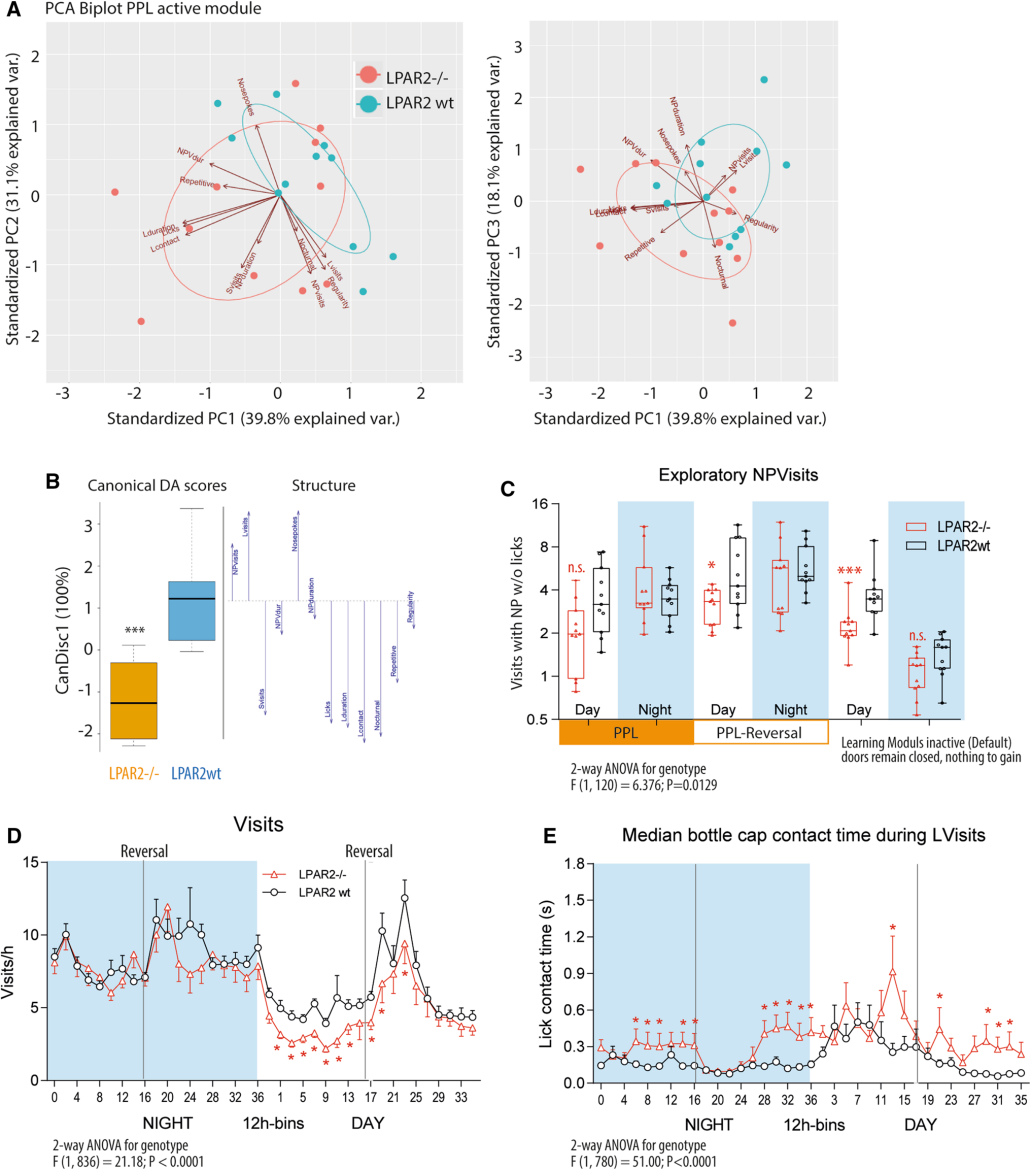
Behavior of wild-type (LPAR2 wt) and LPAR2^−/−^ mice in the IntelliCage Mice had to learn to prefer one corner and one side in this corner to get access to one specific drinking bottle. The groups comprised *n* = 12 female mice, with an age at onset of IntelliCage experiments of 50–60 weeks and 58–70 weeks in controls and knockouts, respectively. The time course of basic behavioral parameters and sequence of the IntelliCage experiments are shown in Suppl. Figure 2. **a** Discriminant Principal component analysis (PCA) biplots for the first two PCA components. The blue arrows show the loading vectors of the behavioral parameters, the dots are the score means and the ellipse shows the 95% confidence interval. Groups differed mainly in licking parameters. **b** Canonical Discriminant Analysis scores and structure. CanDisc1 explained 100% of the variability. Licking behavior (Licks, contact times, licking duration) was lower in LPAR2^−/−^ mice; whereas, exploratory behavior (visits with NPs w/o licks, Nosepokes) was stronger in LPAR2 wt mice. Scores were compared with a two-tailed unpaired *t* test, ***P < 0.001. **c** Box/Scatter plots showing exploratory NPVisits (visits with nosepokes without licks) during the different learning modules, which were active 2 × 3 h per day. In between, default modules ensured that doors remained closed, and nothing was to gain. LPAR2^−/−^ mice made fewer NPVisits during the day, particularly during default modules. **d** Time courses of visits/h of 12 h bins at night and daytime during the place preference learning (PPL) and PPL–reversal (PPL-rev) tasks. LPAR2^−/−^ mice made significantly fewer visits during the day, particularly in the PPL task. Activity increased in both groups upon reversal of the correct corner to the opposite side. **e** Circadian phased behavior showing the time courses of 'median licking contact times' during PPL and PPL-rev. LPAR2^−/−^ mice made fewer visits as shown in **d**, but made more and longer-lasting lickings during their visits. As a result, total licks are normal or increased during learning periods (Suppl. Figure 2), in which licking is a measure of success Data were compared with two-way ANOVA for "module" X "genotype" or "time" X "genotype" and in case of significant results, genotypes were subsequently compared using two-tailed unpaired *t* tests using an adjustment of *P* according to Šidák. Asterisks indicate significant differences, *< 0.05, **< 0.01, ***P < 0.0001

Activity parameters in the IntelliCage revealed a "lazier" behavior of LPAR2^−/−^ in terms of visits and NPs during adaptations and simple tasks, in which licking access was easy and did not require stronger efforts (Suppl. Figure 2). LPAR2^−/−^ mice only speeded up during difficult spatial sequence learning and delayed response-learning tasks, when this effort was imperative to maintain licking success (Suppl. Figure 2). Principal component analysis and canonical discriminant analysis of IntelliCage behavioral parameters during a place preference learning task (Fig. 2a, b) revealed that the separation of the genotypes was mainly based on differences in parameters of goal-directed licking activity (licks per visit, licking time and contact time), which were increased in LPAR2^−/−^ mice; whereas, mere exploration was reduced, including "unnecessary" visits and nosepokes that were not coupled with licking (Fig. 2c). Further detailed analyses of time courses of visits and licks (Fig. 2d, e) revealed that the corner visiting activity was significantly reduced during daytime but normal at night. LPAR2^−/−^ mice compensated the "lazier" daytime behavior by making more licks per correct visit, particularly during the most difficult reversal learning periods (Fig. 2e). Hence, they achieved the drinking goal with less effort, so that the total daily number of licks was normal or mildly increased throughout the experiments except in Free Adaptation when licking is not a measure of success but part of the exploratory behavior (Suppl. Figures 2, 3E). The reduction of non-goal-directed exploration is clearly revealed in the actograms and cosinor analyses during free adaptation (Suppl. Figure 3), and was also particularly evident in the 'visits with nosepokes but without licks' (NPVisits) during default modules, i.e., when doors remained closed and nothing was to gain (Fig. 2c). It is of note that LPAR2^−/−^ mice were not overweight at any time up to old age (Suppl. Figure 4). Hence, more efficient resting during the day did not cause obesity, but resulted in better memory consolidation and learning performance in the night.

### Faster success with fewer trials

**Fig. 3:**
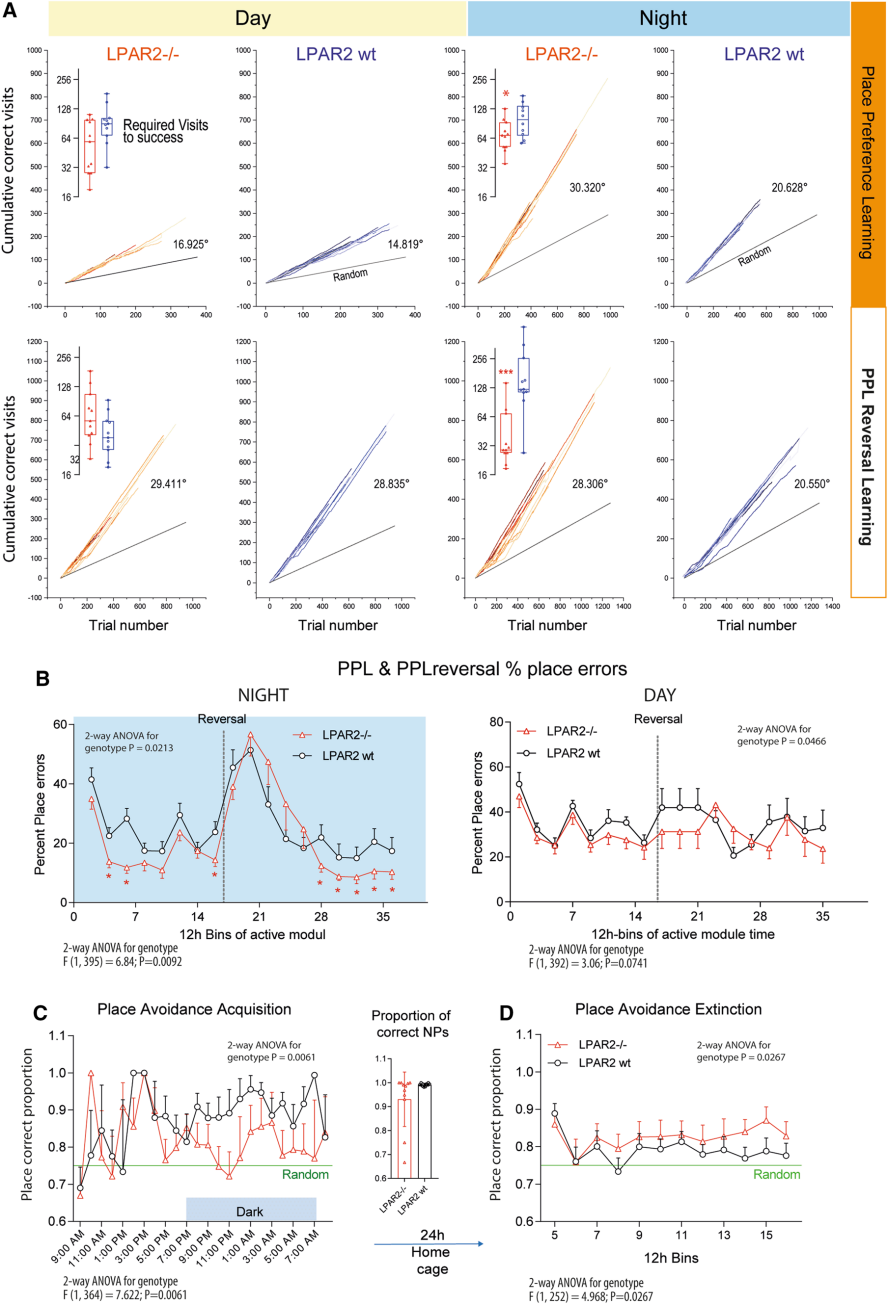
Learning and memory in reward and avoidance-based learning tasks in the IntelliCage in wild-type (LPAR2 wt) and LPAR2^−/−^ mice (mice of Fig. 3). **a** Sequential probability ratio learning plots of place preference learning (PPL) and REVERSAL learning (PPL-rev) tasks show the cumulative number of correct visits relative to the trial number. The steepness of the curves and angle versus the random line indicates the velocity of learning. The inserts show the number of visits, which were required to reach the criterion of success, which was set to 0.35 (random expectation 0.25). Type 1 and 2 errors were set to 0.05. The 'visits to criterion' were compared with two-way ANOVA and subsequent *t* tests using a Šidák adjustment of multiplicity. *P < 0.05, **P < 0.01, ***P < 0.001. **b** Time course of the proportion of place errors (wrong visits relative to all visits) during active modules during the night and day in the PPL and PPL-rev . **c** In place avoidance acquisition (PAL) the task was to avoid one corner, in which nosepokes on both sides were punished with an airpuff and doors remained closed. PAL was run for 24 h. The time courses show the proportion of correct visits (random 0.75) during acquisition. LPAR2^−/−^ mice did not avoid visiting the punished corner, but learnt not to make NPs in this corner. The insert shows the proportion of correct NPs. **d** After a home cage interval of 24 h, avoidance extinction was assessed. Nosepokes opened all doors without punishment or restriction. Red LED reminded of the previously punished corner. The time courses show the proportion of correct visits (random 0.75) during extinction. Loss of memory for the "bad corner" would result in random corner usage Time course data were compared with two-way ANOVA for "time" X "genotype" and genotypes were subsequently compared using two-tailed unpaired *t* tests using an adjustment of *P* according to Šidák. Asterisks indicate significant differences, *< 0.05

Comparison of the cumulative learning curves and number of trials needed to reach the criterion of success (Fig. 3a) shows that indeed, daytime learning was equal, but in the night, LPAR2^−/−^ mice needed significantly fewer trials to achieve success (inserts), and the learning curves were steeper with a higher probability of learners. Comparison of the proportion of place errors (i.e., wrong corner visits, Fig. 3b) over time clearly shows the significantly higher accuracy of LPAR2^−/−^ mice during the night. However, in the first 2.5 days following corner reversal, LPAR2^−/−^ mice did not outperform the controls showing that although reward-directed learning was faster than in controls, this advantage did not apply to the initial reversal, which is supposed to involve muscarinic hippocampal circuits [30] and its functional interactions with the frontal cortex [31]. It is of note that the circadian rhythms were synchronized during learning tasks because the design of the task defined the times, in which a correct nosepoke resulted in door opening (module active, 11–2 a.m. and 11–2 p.m). Hence, all mice had to adhere to this design and the night/day differences did not arise from changes of the acrophase.

### Maintenance of curiosity during airpuff avoidance

Reward-based learning depends on the appetitive drive. In experiments addressing sugar-liking (Suppl. Figure 4), LPAR2^−/−^ were less attracted by reward and sweet taste, suggesting that the appetitive drive in the IntelliCage PPL tasks might have been lower. Therefore, we additionally assessed learning by punishment consisting in an airpuff upon nosepoking in the wrong corner. Normally, mice almost completely stop visiting this corner after receiving one or few airpuffs. The proportion of correct visits reaches 90–100% within a couple of hours (Fig. 3c). In contrast to this normal avoidance behavior, LPAR2^−/−^ mice maintained visiting the respective corner but without making nosepokes (insert of Fig. 3c), suggesting that they were curious but cautious enough not to make a nosepoke. In the extinction period, all mice rapidly lost avoidance, but LPAR2^−/−^ mice regained a dislike of the "bad corner" suggesting stronger attention to the LED, which still reminded of the corner function during acquisition (Fig. 3d). The time courses during acquisition and reversal differed significantly between groups (ANOVA results in Fig. 3c, d). Avoidance learning crucially depends on hippocampal functions [32]. The contextual LED avoidance after airpuff is reminiscent of fear conditioning, which is typically ascribed to the hippocampus [33–35].

### Better performance in 5-choice serial reaction task in LPAR2^−/−^ mice: higher attention

**Fig. 4:**
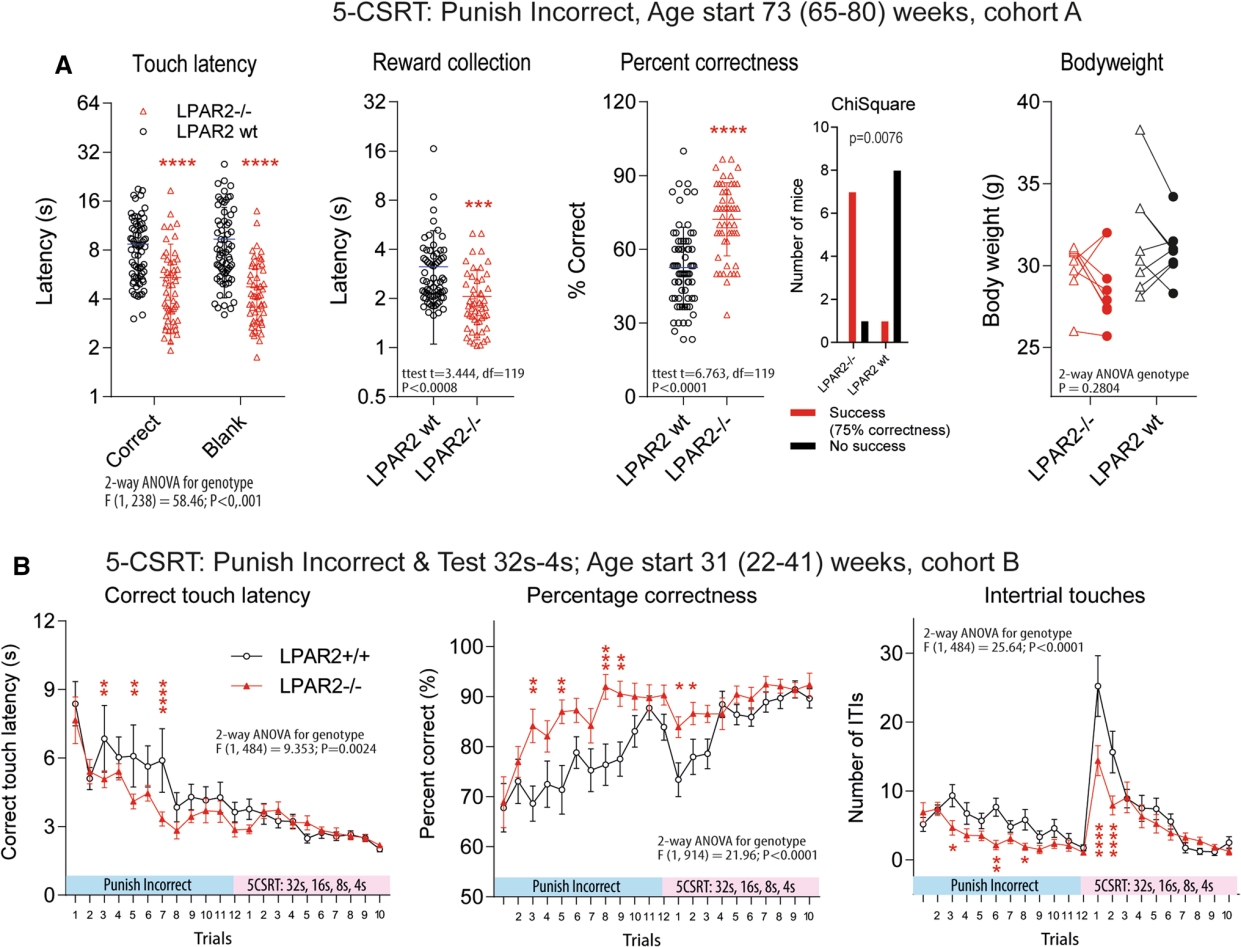
Learning, attention and reversal learning in a touchscreen five-choice serial reaction time task (5CSRT) and paired discrimination and reversal (PD and PD-Rev) task in wild-type (LPAR2 wt) and LPAR2^−/−^ mice. Groups comprised of 8 male mice per group at an age of 65–80 weeks (**a**) or of 12 female mice per group at an age of 20–40 weeks (**b**–**d**) at the onset of touchscreen experiments. For touchscreen experiments, mice were subjected to a 10% reduction diet to increase the learning motivation. The sequential trainings consisted in habituation, 'must touch', 'must initiate' and 'punish incorrect' before entering the 5CSRT basic trial or the PD trial. **a** Touch and reward collection latencies and percentage of correct responses during the 'Punish Incorrect' training period of the 5CSRT. Data were compared with two-tailed, unpaired *t* tests, ***P < 0.001, ****P < 0.0001. The body weights show the efficacy of the 10% reduction diet. **b** Time courses of the correct touch latency, percentage of correct responses and number of inter-trial touches, the latter reflecting the impulsivity. The data show the responses during the Punish Incorrect training trials and the subsequent 5CSRT trials with decreasing stimulus duration (3 × 32 s, 3 × 16 s, 3 × 8 s, 1 × 4 s) Time course data were compared with two-way ANOVA for "time" X "genotype" and genotypes were subsequently compared using two-tailed unpaired *t* tests using an adjustment of *P* according to Šidák. Asterisks indicate significant differences, *P < 0.05, **P < 0.01, ***P < 0.001

To further address attention, we used the 5CSRT touchscreen task, which is specifically designed to test responses to short visual stimuli. The performance of LPAR2^−/−^ mice was significantly superior to the controls, both, in terms of velocity and accuracy, and the number of mice reaching the criterion of success (Fig. 4a). The time courses, done with a second set of younger animals (cohort B, Fig. 4b), revealed that the controls eventually caught up with the LPAR2^−/−^ mice but needed more trials and made more erroneous premature inter-trial responses, suggesting better impulse control of LPAR2^−/−^ mice, which is supposed to depend on hippocampal functions [36]. Experiments were done during daytime possibly leading to an underestimation of the learning differences. In addition, the lower appeal for sweet liquid in LPAR2^−/−^ mice (Suppl. Figure 4B) might have limited their motivation. Indeed, the loss of body weight under the motivation diet was more homogenous and somewhat stronger in LPAR2^−/−^ mice (Fig. 4a for cohort A, Suppl. Figure 4C for cohort B) suggesting that they were less interested in food.

### No difference in paired discrimination learning and reversal learning

In contrast to the superior performance in the 5CSRT, LPAR2^−/−^ mice had no advantage in the paired discrimination and reversal touchscreen task, which relies on recognition and discrimination of visual objects and does not require speed and high attention to short stimuli (Suppl. Figure 4D). The performance in the discrimination task is supposed to depend on glutamatergic and muscarinergic cortical–hippocampal circuits [37–39]. Again, genotype differences might have been underestimated because of the lower sugar appeal for LPAR2^−/−^ mice (Suppl. Figure 4B), and overall low motivation of the very old mice, which were 80–90 weeks old during these experiments.

### Reduction of neuronal activity markers in LPAR2^−/−^ mice

**Fig. 5:**
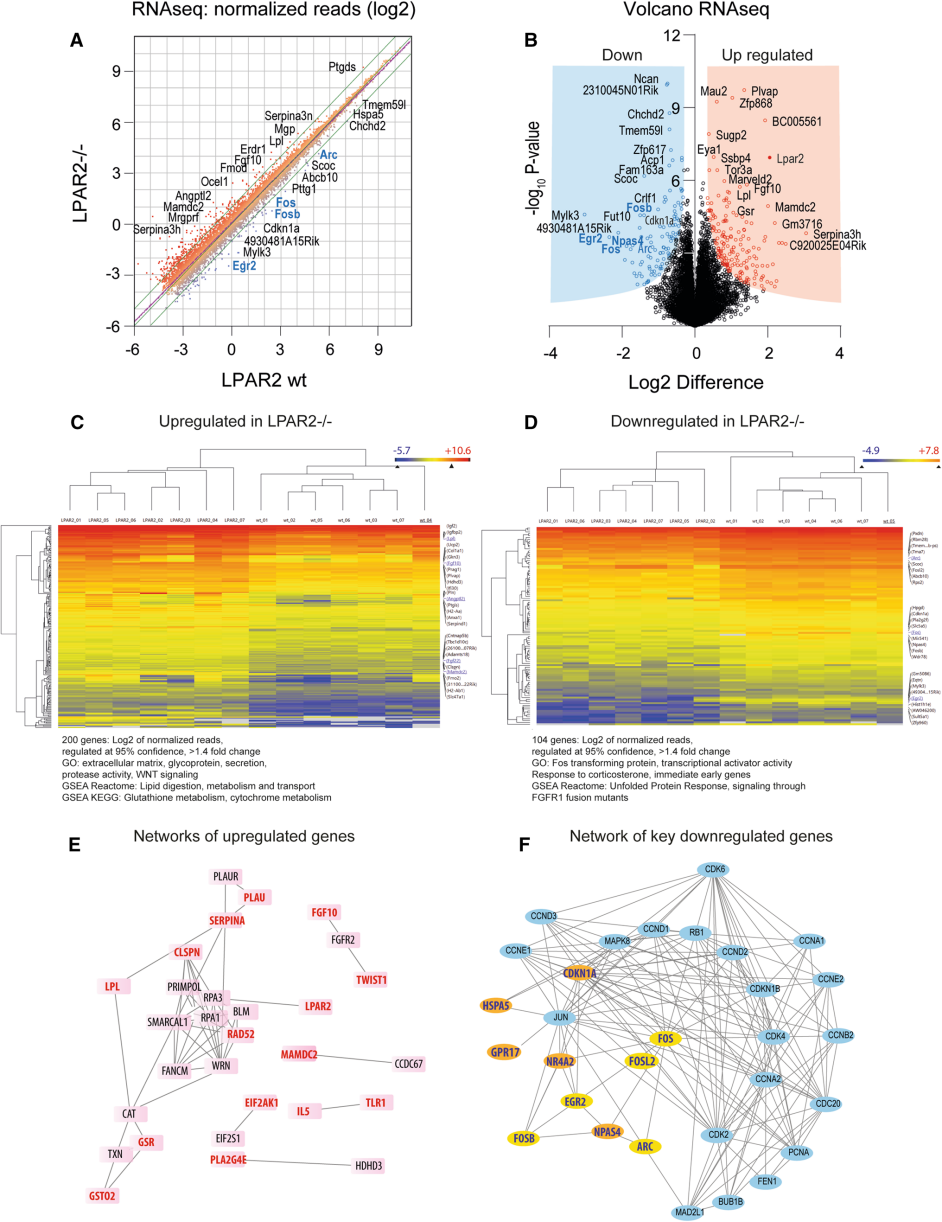
RNA sequencing of the hippocampal dentate gyrus of naïve LPAR2^−/−^ versus LPAR2 wt mice. **a** Log2 scaled scatter plot of the group means of Deseq2 normalized reads of *n* = 7 mice per group. The dotted line is the best-fit line (linear regression) and the green lines show the twofold range. Some outmost dots are labeled with the gene name. **b** Volcano plot showing the group difference of log2 transformed reads on the *x*-axis versus the –log10 of the *t* test *P* value of the *y*-axis. Some prominent candidate genes are labeled with the gene name. **c**, **d** Heatmaps and dendrograms of Euclidean hierarchical clustering of genes and of experiments for significantly up- or downregulated genes (at 95% confidence) with at least 1.4-fold change. The color scale ranges from − 2.5 to + 2.5 SD. Major significant GO terms for biological process (BP) and cellular component (CC) and pathways are given underneath the heatmaps. GSEA ranked leading edge genes in Suppl. Figure 5. **e**, **f** Networks of most relevant regulated genes according to ranked lists based on *P* value, fold change and abundance. The networks were generated using STRING and MIPPIE and further adapted in Cytoscape. Primary hits are highlighted by bold lettering

To assess biological correlates of the relaxed nature and maintenance of "youthful" neuronal excitability provided by LPAR2 deficiency, we analyzed the hippocampal transcriptomes. RNAseq analyses using dentate gyrus of each 7 naive mice per group revealed a downregulation of neuronal activity-related genes in LPAR2^−/−^ mice including cFos, FosB, Arc, Fosl2, Egr2, Npas4, Nr4a1 and Nr4a2 and Creb1, clearly showing a biological correlate to lower baseline neuronal activity (Fig. 5a, b), with a clear-cut clustering of mice according to the top regulated genes (Fig. 5c, d). The leading edge genes according to GSEA analysis (top 50 up- and down-regulated in Suppl. Figure 5) and networks of top candidates (Fig. 5e, f) agree with GO enrichment analyses. Downregulated genes pointed to lower ER stress and reduced responses to corticosteroids, the latter supporting the view of a more "relaxed" phenotype of LPAR2^−/−^ mice. Enriched GO terms of upregulated genes suggest an increase of secreted proteins and extracellular matrix components and a pathway enrichment for 'lipid digestion, metabolism and transport' (including genes like lipoprotein lipase, phospholipases, prostaglandin receptor and synthase, LDL receptor, APOA1 and APOE), and of glutathione metabolism.

### LPAR2 antagonist leads to more "relaxed" goal-directed behavior with better learning outcome

**Fig. 6:**
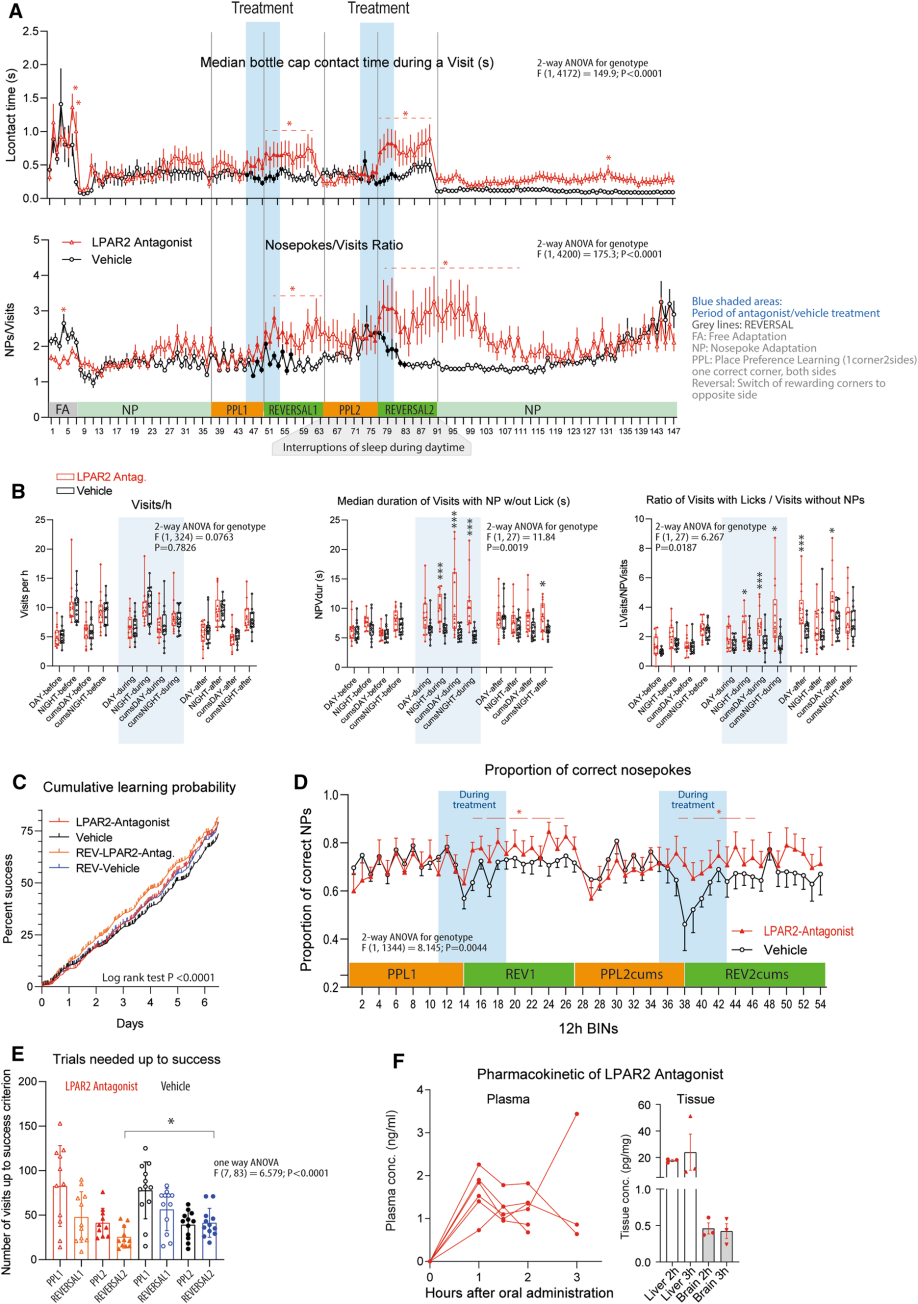
IntelliCage activity and learning in mice treated with LPAR2 antagonist versus vehicle. Mice were treated with 50-µg LPAR2 antagonist in 2-g cornflakes (*n* = 13) or vehicle cornflakes (*n* = 12) once daily in the morning during the blue shaded periods, also indicated with filled red and black symbols. Mice were 74–75 weeks old at the onset of observation time. **a** Time courses of key behavioral parameters. Mice were adapted with free adaptation (FA) and nosepoke adaptation (NP) and put on a mild restriction diet (3 g of pellets per mouse and day) to increase the appetite for the medication-cornflakes. Place preference learning (PPL1) was started and cornflakes (without drug) were introduced as morning meal. The treatment period encompassed the last two days of PPL1 (or PPL2) up to the third day after Reversal of PPL1 (or Reversal PPL2) (filled symbols, blue shaded areas). Reversal refers to the switch of the rewarding corner to the opposite site. The first learning and reversal (PPL1 and Reversal) was without any disruptions, and the second PPL2 and Reversal was done with daily disruptions of daytime sleep in random order explained in Suppl. Table 4. The data are means ± SEM. Differences between groups were assessed with two-way ANOVA. Asterisks point to significant time points or periods for the between-subject factor "group" (*P* not adjusted for multiple time point assessment). Dashed lines indicate that not all consecutive individual time points were significant. Period averages and statistics are shown in B. The tasks and daytime sleep disruptions during PPL2/REVERSAL2 are explained in Suppl. Table 2 and Suppl. Table 4, respectively. **b** Box/scatter plots showing the average behavior during the specified periods in mice treated with LPAR2 antagonist or vehicle. The box shows the interquartile range, the line is the median, whiskers show minimum to maximum. The dots show results of individual mice. Before, during and after refer to the treatment period. 'cums' refers to the chronic unpredictable stress evoked by disruptions of daytime sleep. Data were compared with two-way ANOVA followed by *t* tests to compare treatment groups and the interaction of "time × treatment". *P* values were adjusted according to Sidak, ***adjusted *P* < 0.001, *adjusted *P* < 0.05. **c** Sequential probability learning plots showing the cumulative percentage of correct visits relative to the start time of the trial. The steepness of the curves indicates the velocity of learning. Each spike is an event (correct visit). The curves were compared with linear regression statistics. **d** Time course of the proportion of correct nosepokes during place preference learning and REVERSAL periods. The data are means ± SEM. Differences between groups were assessed with two-way ANOVA. Asterisks point to significant time points or periods for the between-subject factor "group" (*P* not adjusted for multiple time point assessment). Dashed lines indicate that not all consecutive individual time points were significant. **e** Learning performance: Trials needed to reach the criterion of success, set at 35% correctness of corner visits. The random correctness is 25%. Data were compared with one-way ANOVA followed by post hoc *t* tests to compare selected groups (i.e., the treatment groups for each learning task) using an adjustment of *P* according to Šidák. Asterisks show significant differences with *P < 0.05 as indicated. Learning speed increased with each corner reversal. LPAR2 antagonist-treated mice succeeded with fewer trials during the most difficult REVERSAL2 period under 'circadian stress'. **f** Plasma concentration time courses and tissue concentrations after per oral administration of 50-µg LPAR2 antagonist 1, CAS No.1017606-66-4, in 10% DMSO/10% sucrose-soaked cornflakes. Each line/scatter is a mouse. *FA* free adaptation, *NP* nosepoke adaptation, *PPL1* place preference learning (one correct corner, both sides) without stress, *PPL2* place preference learning with daytime sleep disruptions causing mild stress, *REVERSAL1 and* *REVERSAL2* correct corners switched to opposite side of the cage

To assess the therapeutic implications of our observations in LPAR2^−/−^ mice, we performed IntelliCage experiments before, during and after daily oral administrations of an LPAR2 antagonist versus vehicle in old mice (Fig. 6, Suppl. Figure 6). The antagonist was administered orally during place preference learning periods without/with disturbances of daytime sleep, and the treatment periods encompassed the critical REVERSAL learning periods. Time course analyses of basic behavioral parameters revealed similar visits, nosepokes and licks in LPAR2 antagonist and vehicle groups (Suppl. Figure 6A), but upon treatment onset, mice receiving the antagonist spent significantly more time on individual visits (longer duration of 'visits with nosepokes with/out licks'), and increased the numbers of visits with licks (LVisits) but reduced visits without licks (NPVisits) (Fig. 6a, period-averaged behavior in Fig. 6b). The behavior suggested that LPAR2 antagonist-treated mice were more relaxed and focused on efforts with rewarding probability. Notably, goal-directed behavior requires inhibitory input to the ventral hippocampus [40, 41], which might be less effective with age-associated hyperexcitability.

After a 14-day period of unpredictable disturbances of daytime sleep causing mild stress, mice which had received the LPAR2 antagonist in the sleep-disruption/stress period regained circadian rhythms faster in the post-stress period (Suppl. Figure 6b). The more relaxed behavior in LPAR2 antagonist-treated mice was associated with faster learning (significantly steeper learning curves, Fig. 6c) and significantly higher proportion of correct nosepokes (Fig. 6d). They also needed fewer trials to reach the criterion of success (10% above random, i.e., 35% correctness) in the REVERSAL period under stress (Fig. 6e). Pharmacokinetic data of plasma and brain concentrations of the LPAR2 antagonist confirmed that the drug was rapidly bioavailable after oral administration and passed the blood–brain barrier (Fig. 6f).

## Discussion

Cortical and hippocampal glutamatergic transmission is regulated by synaptic LPA generation and signaling, leading to presynaptic LPAR2-mediated augmentation of glutamate release probabilities. The enhancement is counteracted by postsynaptic PRG1-mediated LPA scavenging [3, 10]. Loss of PRG1 results in neuronal hyperexcitability and a schizophrenia-like phenotype [2, 10]. The present study shows that deficiency of the presynaptic LPAR2 is associated with (1) prevention of age-associated hippocampal hyperexcitability and enhancement of hippocampal LTP in middle-aged mice, (2) higher accuracy and response velocity in tasks requiring high attention, (3) lower mere exploratory activity during non-active daytime and (4) lower expression of neuronal activity marker genes in resting naïve animals. Importantly, therapeutic once-daily oral LPAR2 inhibition recapitulated the phenotype with increased resting and goal-directed behavior, and improved learning under stress.

It is well accepted that hippocampal LTP is driven by glutamate, and intact CA3–CA1 synapses are essential for recall [25]. While LPAR2 did not affect LTP formation in young animals, LPAR2 deficiency increased synaptic efficacy upon high-frequency stimulation in slices of middle-aged mice. Since our data show that LPAR2 deficiency prevented age-related hippocampal hyperexcitability, the observed LTP enhancement in LPAR2 knockout mice points to secondary beneficial mechanisms resulting from life-long shielding against periods of glutamatergic "overflow", which increase upon aging [21] or in prodromal phases of neurodegenerative diseases [17]. This is in line with data from primates and rats showing that hippocampal neuronal hyperexcitability is a feature of the aging brain [20, 21] and might lead to an age-related vulnerability of hippocampal circuits that are associated with cognitive impairments as observed in Alzheimer’s disease [42, 43]. Previous data show that lower neuronal excitability directly correlates with better memory performance in rats [44] and humans with mild cognitive impairment [45, 46]. Particularly, tasks requiring high attention need an inhibitory tone to avoid too much distraction by environmental stimuli. In line with this idea, LPAR2^−/−^ mice were less active in times when high action was either unnecessary (Free Adaptation in IntelliCage), or futile (default modules in IntelliCage—doors remained closed). Hence, they were reluctant to explore without need and utilized correct visits more efficiently in that they increased the licks per visit and the licking times. The bottom line during learning periods was that they achieved normal daily licking numbers with far less effort. They also avoided the exploratory over-drinking during Free Adaptation. We conclude that lowering of glutamatergic excitability increases the use of resting periods, reduces distraction by concurrent environmental stimuli, reduces unnecessary exploratory behavior in favor or goal-directed behavior and allows for better selective attention and better performance in tasks requiring high attention and fast responses to visual light/LED stimuli. The behavior of LPAR2^−/−^ mice has some similarity with mouse behavior under methylphenidate in models addressing attention or sensory processing [47–49]. Notably, glutamate overflow leads to attentional deficits [50] and the 5CSRT touchscreen task has indeed some predictive validity for selecting drugs effective for treating attention-deficit/hyperactivity disorder [51].

Mere exploration behavior and erratic hyperactivity is negatively associated with hippocampal neurogenesis [52, 53], suggesting that the observed quieter phenotype of LPAR2^−/−^ mice reflects stronger renewal. Readouts of locomotion such as travel paths in maze tests were all normal and did not suggest that stronger daytime resting was caused by any motor impairment. Further, zero-maze behavior would agree with lower anxiety of LPAR2^−/−^ mice and argue against an anxiety-dependent suppression of corner visiting activity.

In touchscreen experiments, the responses were faster, more accurate and LPAR2^−/−^ mice made fewer premature inter-trial touches and fewer perseverative touches (i.e., touches after feedback), the latter suggesting a lower level of attentive impulsivity [50, 54]. Hence, attenuation of the LPAR2-mediated glutamatergic synaptic enhancement appears to improve resting and attention by reducing excitability and distraction. Therefore, LPAR2 antagonism may be useful in attentional deficit disorders or restlessness, particularly in aged individuals. In support of this therapeutic idea, oral once-daily administration of an LPAR2 antagonist increased the duration of visits and the time the mice spent licking during such visits. This more relaxed behavior led to a higher accuracy and higher rate of success, particularly during and after the most difficult last Reversal Learning under stress. The differences was also notable in the first reversal, but got stronger with each reversal. The hippocampus is necessary to anticipate the occurrence of reversals, and disruptions of the hippocampus can render behavior habitual and inflexible [55], suggesting that the LPAR2 antagonist helped to maintain flexibility. In addition, LPAR2 antagonist-treated mice had a faster reestablishment of circadian rhythms in the post-stress/post-treatment period. It is of note that vehicle-treated mice but not the LPAR2 antagonist-treated mice showed a mild loss of licking behavior in the post-stress period, which is often interpreted as a readout of depression-like behavior and has been associated with a stress-evoked suppression of adult neurogenesis in the hippocampus [56].

The data would agree with a protective effect of LPAR2 antagonism in the context of stress-evoked depression and age-associated restlessness, which are typical findings in conditions related to hippocampal hyperexcitability upon normal aging [20] or in neurodegenerative disorders such as Alzheimer's disease [57]. Tasks that require more attention increase the need for resting periods, suggesting that efficient resting is required to filter incoming stimuli according to their relative salience.

Our data suggest that the LPAR2/PRG1 system plays an important role in this filtering process, PRG1 by regulating the synaptic LPA content and LPAR2 by translating the LPA signal to glutamate release. During evolution, sophisticated forms of cognition such as operant learning and selective attention evolved in parallel with neural mechanisms that support sleep, i.e., stimulus suppression and behavioral quiescence [58, 59]. Selective attention and sleep both require a suppression of the outside world, on either a selective or a global level. Extensive evidence suggests that sleep facilitates learning and memory consolidation in mammals [60–63] suggesting that the learning superiority of LPAR2^−/−^ mice during the night may result in part from efficient resting during the day. However, we did not directly assess sleep and wakefulness in the present study. Actograms provide only an indirect view on activity patterns and long-term EEG recordings in freely moving mice in the IntelliCages would be needed to assess effects on LPAR2 antagonism on sleep patterns relative to the learning performance.

The benefits of LPAR2 deficiency appear to be cumulative during life, because the increase of LTP only occurred above 30 weeks of age but was not obvious in young adult animals, which agrees with the behavioral experiments, which were all performed in mice beyond youth up to old age. The influence of age was not a primary outcome variable because the long duration of behavioral testing precluded a repetition of all tasks at any age. Touchscreen 5CSRT experiments were done with a cohort of male old mice and a cohort of female middle-aged mice. The results were consistent and reproducible and argue against gender as relevant confounder. A further limitation is that most of the behavioral tests, except for IntelliCage observations, were done during daytime and it is conceivable that we have underestimated some LPAR2^−/−^ mice learning advantages, which got more evident during the night.

From the mouse point of view, the reduction of non-goal-directed exploratory behavior is energy saving and efficient. However, translated to humans, blocking LPAR2 to increase attention might lead to a loss of enjoyable daily distractions and exciting stimuli, and possibly increase monotony in life. Indeed, LPAR2^−/−^ mice were less interested in sweet rewards, obviously crucial for body weight maintenance with lower activity, but possibly for the expense of losing pleasure. Evolution obviously decided to keep LPAR2, although cognitive advantages of not having LPAR2 in mice outweighed the disadvantages, which may hold true in modern life where information overflow is a frequent cause of stress and disease. Insofar, LPAR2 inhibition might have therapeutic value.

## Methods

### Mice

LPAR2 knockout mice (LPAR2^−/−^) are deficient of the second half of exon 2 of *lpar2* [64]. LPAR2^−/−^ mice were maintained as homozygous colony in parallel with a colony of the controls, which have a mixed C57BL/6 and 129/SvJ genetic background. Experiments were performed with age- and gender-matched 7–13 animals per genotype at 20–88 weeks, specified in the figure legends. The behavioral experiments were done with two consecutive cohorts of old male mice and middle-aged to old female mice. The schedule of behavioral tasks including sample sizes, gender and age is presented in Suppl. Table 1. For IntelliCage experiments involving treatments with an LPAR2 antagonist (CAS 1017606-66-4, MedChemExpress, New Jersey, USA) versus vehicle, we used 24 female mice (EGFL7^−/−^ versus wildtype), which were 74–75 weeks old at the start of the experiments, plus two C57BL6/J mice at 39 weeks to fill places in the IntelliCage. Mice deficient of epidermal growth factor-like 7 (EGFL7) [65] are mildly hyperactive at advanced ages and were, therefore, particularly useful for the assessment of LPAR2 antagonist effects. For analysis of the drug effects, both genotypes were pooled to increase power. The two younger mice were not included.

Naïve mice were used for electrophysiology and RNA sequencing experiments.

Mice were allowed to acclimatize to the experiment rooms, cages or mazes before starting experiments. They had free access to food and water, and they were maintained in climate-controlled rooms at a 12-h light–dark cycle. The experiments followed the “Principles of laboratory animal care” (NIH publication No. 86-23, revised 1985). They were approved by the local Ethics Committee for animal research (Darmstadt, Germany), adhered to the guidelines of the International Association for the Study of PAIN (IASP) and were in line with the European and German regulations for animal research and the ARRIVE guidelines.

### Barnes Maze spatial learning and memory

Old male mice were used for analysis of avoidance-based spatial learning (88–97 weeks LPAR2^−/−^, *n* = 7; 89–93 weeks control mice, *n* = 8) with a classical Barnes Maze (TSE, Bad Homburg, Germany). Performance in the Barnes maze is supposed to be contributed by "anxiety cells" in the ventral CA1 region and "place cells" in the dorsal CA1 [34]. Flexible reversal learning requires both the dorsal and ventral hippocampus and their functional interactions with the prefrontal cortex [31, 66]. The maze was divided into five zones: center, target (i.e., rewarding box), opposite, positive and negative. Mice were randomly assigned to one of the four different positions for the escape cage. The protocol consisted of three phases: habituation, learning, and reversal learning. In the habituation phase, mice were set under a plastic cylinder for 30 s in the middle of the maze, and were then directed to the target hole, where they were allowed to enter the shelter within 3 min. If not, they were nudged into it and allowed to stay there for 1 min. The habituation was done for 3 days with 3–5 trials per day. In the initial learning phase (3 days, 1 trial each), mice were allowed to freely explore the maze for 5 min to find and enter the target hole. In the subsequent Reversal Learning phase (3 days, 1 trial each) the target box was moved to the opposite side of the maze. EthoVision XT 11.5 software (Noldus, Wageningen, The Netherlands) was used for video tracking and analysis. Trial duration, distance moved, velocity, visits and cumulative duration in each zones were recorded.

### Social cognition and memory

The social discrimination task assesses social cognition and memory according to standard protocols. The recognition of social novelty involves dopaminergic systems in the prefrontal cortex [67]. For this experiment 27–40-week-old female LPAR2^−/−^ mice (*n* = 13) and 20–26-week-old female control mice (*n* = 12) were used. C57BL6 mice (5 weeks old) were used as social stimuli. The box consisted in three chambers of equal sizes (14 × 19 cm). The middle chamber was connected to the outer chambers by doors, which can be closed. A cylindrical enclosure was placed into the corners of each outer compartment, and mice were habituated to the environment one week before test start. At the experiment day, mice were acclimatized to the middle chamber for 5 min with closed doors. The doors were then opened and mice allowed to explore the chambers and enclosures, one empty, the other with a stimulus mouse for 10 min. Subsequently, a second mouse was added to the still empty enclosure allowing assessment of social novelty recognition, again for 10 min. A wild-type mouse is normally more interested in a social partner relative to an empty compartment (social cognition), and spends more time with a novel partner relative to a familiar one (social memory). The trials were recorded with a video camera and analyzed with VideoMot 2 software (TSE Systems GmbH, Bad Homburg, Germany).

### Elevated zero maze

The elevated zero-maze measures behavioral correlates of anxiety, which is supposed to involve circuits of from the prefrontal cortex to the amygdala [68] and its signaling to hippocampus [34]. The maze has a diameter of 60 cm, a 5-cm-wide circular corridor, 16-cm-high walls and is 60 cm above the floor. For the experiment 28–41-week-old female LPAR2^−/−^ mice (*n* = 13) and 20–26-week-old female control mice (*n* = 12) were used. EthoVision XT 11.5 (Noldus, Wageningen, The Netherlands) 3-point tracking software was used to assess locomotion, velocity, time in zones, visits to zones and poking into open zones. Tracking started immediately after placing the test mouse into one of the two closed semicircles and lasted for 5 min. Mice were tested once without prior habituation, except for the room adaptation.

### IntelliCage

The IntelliCage (NewBehavior AG, Zurich, Switzerland) [32, 69–72] consists of four operant corners, each with two water bottles, sensors, light-emitting diodes (LEDs) and doors that control the access to the water bottles. The system fits into a large cage (20 × 55 × 38 cm, Tecniplast, 2000P). Four triangular red shelters (Tecniplast) are placed in the middle to serve as sleeping quarters and as stands to reach the food. The floor is covered with thick bedding. Mice are tagged with radio-frequency identification (RFID)-transponders, which are read with an RFID antenna integrated at corner entrance. Inside the corners, there are two holes with water bottles, which can be opened and closed by automated doors. Mice have to make nosepokes (NP) to open the doors for water access. The IntelliCage is controlled by a computer with IntelliCage Plus software, which executes pre-programmed experimental tasks and schedules. The numbers and duration of corner visits, nosepokes, and licks are automatically recorded without the need for handling of the mice during the recording times.

### IntelliCage behavioral tasks

IntelliCage tasks address a number of different aspects of cognition as well as circadian rhythms and social interactions, and were run sequentially. The tasks are described in Suppl. Table 2, and abbreviations of behavioral parameters are summarized in Suppl. Table 3. The tasks followed established protocols [32, 69, 70, 72, 73] or were new designs. For this experiment, 58–72-week-old female LPAR2^−/−^ (*n* = 11) and 51–57-week-old female control mice (*n* = 12) were used. Twelve mice were housed per cage (6/6 of each genotype).

Mice were adapted to the system for 3 days with free access to every corner, with all doors open, and water and food ad libitum. This free adaptation was followed by 9-day “nosepoke adaptation,” during which the doors were closed, the first nose-poke of the visit opened the door for 5 s and to drink more, the animals had to leave the corner and start a new visit. Mice were then adapted to the “nosepoke drinking session” protocol (DS), in which the nosepoke modul was active only during defined time periods between 11–14:00 h and 23–2:00 hto increase motivational drive. The start of a drinking session was announced by green LEDs for 2 min. Outside of these times the doors remained closed. The adaptation to circadian schedules in the IntelliCage depends on hippocampal functions [74].

In the next “place preference learning” PPL task mice had to learn to prefer a specific corner, where they got the water reward. The PPL modul was only active during the respective drinking sessions. Mild social competition was then introduced by assigning all mice to the same corner. During the daytime drinking session, the left corner was defined as correct and associated with a green LED, and in the night the right side of the corner was correct, which was announced with a red LED upon corner entry. Only the first correct nosepoke opened the door. Incorrect nosepokes had no effect. After conditioning to the corner, PPL reversal learning (PPL–REV) was assessed by switching the rewarding corner to the opposite side. Simple reward-based PPL is not affected by lesions of the frontal cortex or hippocampus, but hippocampal dysfunctions impair the ability to cope with conflicting tasks with additional inherent spatial, visual, temporal or emotional cues [74]. Appetitive learning also involves the central and basolateral amygdala [75, 76]. Salience-based motivation has been assigned to cortical regions and the nucleus accumbens [77, 78]. Flexible spatial reversal learning requires the dorsal and ventral hippocampus and their functional interactions with the prefrontal cortex [31, 55], and is partially lost with disturbances of adult hippocampal neurogenesis [79].

In the “spatial sequence learning” protocol (SSL), mice had to learn that the next rewarding corner was in a clockwise sequence relative to the actual rewarding corner. The switch to the next corner only occurred after visits with licks (Lvisits) but not after exploratory visits without licks ensuring that the mice got the reward. After learning the sequence, the chaining was reversed (SSL-reversal), i.e., now in anti-clockwise rotation. During both SSL tasks, a correct visit was indicated with green LED. Spatial sequence leaning critically depends on an intact hippocampus and its bidirectional connections to the retrosplenial cortex [80].

The “delayed-response-learning” (DRL) task was used to assess impulse control. Mice had to poke a second time at the same door during a visit, with a defined delay. A nosepoke on the opposite door had no effect. The training started with a delay of 4 s (DRL 4 s). Initially, one neutral corner allowed drinking upon one NP as usually, but doors opened in only 50% of the trials. Green LEDs were lit upon entry of a correct corner and switched off after the first correct or incorrect nosepoke. An incorrect nosepoke was defined as a premature second nosepoke on the same door, and mice had to leave the corner and start again with a new visit. The difficulty was increased by closing the neutral corner and subsequently by increasing the delay to 6 s (DRL 6 s). The licking success of some mice dropped to zero in the DRL 6 s, so that a water gel recovery package was provided overnight in both cages. Impulse control in the delayed response-learning tasks is impaired in rodents with hippocampal or cortical lesions; whereas, striatal lesions are well compensated [81, 82].

In “place avoidance learning” (PAL), mice had to learn to avoid one punished corner, which was randomly assigned to each 4 mice. The punishment consisted in an airpuff (∼ 0.8 bar, 1 s) and was coupled with red LED. The avoidance acquisition lasted for 24 h. At completion, mice returned to their home cages for 1 day with water restriction for the last 18 h prior to their return to their IntelliCage for the analysis of the extinction of the avoidance behavior. The water restriction ensured that all mice were equally thirsty and highly motivated to get water. The IntelliCage was not cleaned during the home cage stay to maintain the environmental and olfactory cues. In the following 6 days, extinction of the aversive memory was assessed (PAExt), in which all doors opened in response to a nosepoke and no airpuff was applied. Only the red LED still reminded of the previously 'punished' corner. Conditioned place avoidance in the IntelliCage is sensitive to functions of the hippocampus [32] and reminiscent of fear conditioning in classical foot shock-based tests of hippocampal functions.

### IntelliCage with LPAR2 antagonist treatment

To assess putative therapeutic implications, we assessed the effects of an LPAR2 antagonist in a complex set of IntelliCage experiments. In this experiment, we used female wildtype and EGFL7^−/−^ mice, which were 74–75 weeks at experiment start. EGFL7^−/−^ mice are mildly overactive at this age. Mice were randomly assigned to cages 1 and 2, with balanced numbers per genotype. The LPAR2 antagonist (CAS 1017606-66-4, MedChemExpress, New Jersey, USA) was administered orally once daily every morning in cage 1 at 10 am for 2 × 5 d. The drug (50 µg per mouse) was diluted with 10% DMSO/1% sucrose. For each mouse, 2-g cornflakes were soaked with the solution. Vehicle controls in cage 2 received vehicle cornflakes. Before the start of the drug treatment period, mice were adapted to the cornflakes and set to a mild food restriction diet of 3 g pellets per mouse per day to enhance the appetite for cornflakes. The IntelliCage protocol consisted in 4-day free adaptation (FA), 3-week nosepoke adaptation (NP), 7-day place preference learning and 7-day REVERSAL learning without stress (PPL1 and REVERSAL1), 7-day place preference learning and 7-day REVERSAL learning in combination with disruptions of circadian rhythms (PPL2 and REVERSAL2), and finally again 5 weeks of an unrestricted NP protocol without stress to observe the reinstatement of circadian rhythms. The antagonist treatment started in the PPL1 and PPL2 tasks two days before REVERSAL, and was continued up to 3 days after REVERSAL, resulting in 2 × 10 12-h cycles with direct influence of the antagonist. Disruptions of circadian rhythms consisted in providing bits of food every hour after overnight fast during the day, darkness for 36 h, repeated change of bedding, and/or removal or exchange of houses for short periods during daytime and Light ON/OFF cycles of 2 h (Suppl. Table 4). These stressors were applied in unpredictable order for 14 days (PPL2 and REVERSAL2).

### Taste preference test

To analyze the influence of LPAR2 on taste perception, 52–65-week-old female LPAR2^−/−^ mice (*n* = 10) and 45–51-week-old female control mice (*n* = 12) were used in the taste preference test. Mice were housed two per cage and habituated by providing two bottles of water per cage. Volume intake of both bottles per cage was measured for three days. In the subsequent phase, bottles with the specific taste were placed into the spontaneously preferred corner. Tastes were provided in random order each for 24 h and consisted in water (neutral), 100-mM NaCl (salty), 10-mM HCl (sour), 0.5-mM hydroxychloroquine (HCQ; bitter), 60-mM sucrose (sweet), 60-mM monosodium glutamate (umami), all diluted in tap water. The volume intake was assessed by weighing the bottles daily.

### Touch screen

Bussey–Saksida touchscreen equipment (Campden Instruments Ltd/Lafayette Instruments) was used as described [83]. The trapezoidal operant chamber consists of an infrared touchscreen spanning the wider end of the trapezoid, a perforated floor and a peristaltic liquid reward supply at the narrow end of the trapezoid. Presentation of stimuli, nosepokes on the screen and capture of the behavior with video cameras were controlled by Lafayette ABET II software. The touchscreen trainings and tasks are summarized in Suppl. Table 5.

### Touch screen—5-choice serial reaction time (5-CSRT)

The 5CSRT touchscreen experiments were performed with two consecutive cohorts of old and middle-aged mice. The first cohort consisted in 64–72-week-old male LPAR2^−/−^ mice (*n* = 8) and 64–80-week-old male control mice (*n* = 8); the second cohort were 27–40-week-old female LPAR2^−/−^ mice (*n* = 12) and 20–26-week-old female control mice (*n* = 12). The ages refer to the start of the experiments. For stimulus presentation within the '5-choice serial reaction time' (5CSRT) task, a black Perspex mask with 5 windows was placed in front of the touchscreen. The training steps were adapted from the procedures published by the Bussey and Holmes laboratories [84, 85]. One week prior to the habituation phase, mice were set on a restriction diet with 2–3 g of food pellets per day to reduce the body weight to 90%. The diet was maintained throughout experiments to increase the appetite for the liquid reward, which consisted in 8-μl sweetened condensed milk (Nestlé, Switzerland; diluted 1:4 in tap water). During the training stages, the animals learnt to touch the screen in correct locations to get a tone-coupled reward. In the “Must-Touch” pre-training, the mouse had to touch the screen at any site in response to the stimulus to collect the reward. In the next step “Must Initiate”, the mouse had to trigger the stimulus by poking into the illuminated reward trough. The trigger-to-stimulus delay was 5 s. Criterion under these conditions was defined by a completion of 30 trials within 60 min on two consecutive training days. For the 5CSRT task, a white-square is presented pseudo-randomly in one out of five possible locations, and the mouse has to touch the screen in the correct position. Outside of the lit rectangle, the screen is dark. In the “Punish Incorrect” training, settings were as in Must Initiate but a timeout period of 5 s was triggered if the mouse touched the blank site of the screen outside of the enlightened square. During timeout, the image disappeared and the overhead lighting (~ 60 lx) was turned on for 5 s. The criterion was to complete 30 trials in 60 min with > 75% correct responses. During the 5CSRT testing phase, settings remained identical to Punish Incorrect, but the time of stimulus presentation was progressively decreased (32 s, 16 s, 8 s, 4 s and 2 s). For each stimulus time, mice performed 3 sessions (1 session per day). The 5CSRT task primarily addresses attention, responses to short visual stimuli, spatial discrimination and impulse control. Sustained attention and translation into action in the 5CSRT depends on dopaminergic neurons in Nc. accumbens [86] and GABAergic systems in medial prefrontal cortex [87].

### Touch screen—pairwise discrimination (PD)

The Pairwise Discrimination task was performed with 77–88-week-old male LPAR2^−/−^ mice (*n* = 7) and 77–84-week-old male control mice (*n* = 8) after completion of the 5-CSRT. For stimulus presentation within the ‘Pairwise Discrimination task’, a black Perspex mask with two windows was placed in front of the touch screen. During different training stages, one stimulus (varying white simple pictograms of objects on black background, Campden/Lafayette software) was presented at a time, in one of the two windows. The animals were allowed to habituate to the testing cages and learned to touch the screen on correct locations to elicit a tone-coupled reward, according to the protocol explained above. During test sessions, two novel stimuli were presented in a spatially pseudorandomized order over 30-trial sessions (20-s intertrial interval, ITI) in the absence of overhead lighting. One image was set to correct, the other to incorrect. Responses to the correct stimulus resulted in 8-μl reward. Responses to the incorrect stimulus resulted in a 5-s timeout, coupled with switching the ∼ 60 lx house light on. This was followed by a correction trial. Stimuli remained on the screen until a response was made [39, 88, 89]. The criterion to enter the reversal stage was to complete 30 trials in 60 min with > 75% correct responses, for a minimum of 3 consecutive testing days. During the reversal stage, the previous correct image was set to incorrect and was coupled with overhead lighting for 5 s. On the contrary, the previous unrewarded image was set to correct and elicited the tone-coupled reward supply. The criterion for reversal learning was an average percent correctness of 80% or higher. The performance in the Pairwise Discrimination task involves functioning of glutamatergic and muscarinic systems [39, 90, 91].

### Field potential recordings

Brain slices were prepared from naïve LPAR2^−/−^ and WT mice, which were young (10–16 weeks) or middle-aged to aged (35–81 weeks) at the day of killing. The ages for each experiment are specified in Suppl. Table 6. Mice were anesthetized with isoflurane, decapitated and the brain was rapidly removed and placed in ice cold ACSF (in mM; 126 NaCl, 2.5 KCl, 1.25 NaH_2_PO_4_, 1 MgCl_2_, 2 CaCl_2_, 26 NaHCO_3_, 10 d-glucose). Horizontal slices (400-µm thick) were prepared using a Leica VT1200S vibratome and equilibrated for at least 1 h at 32 °C prior to the experiment. For recordings, slices were transferred to a submersion-type recording chamber at 32 °C and allowed to recover for 30 min after placing of the electrodes. fEPSPs were evoked by stimulation of Schaffer-collateral fibers with biphasic constant pulses (0.2 ms/polarity) at 0.033 Hz using tungsten microelectrodes of 300–500 kΩ. Stimulation strength was adjusted to 30% of the maximum amplitude of the input–output (*I/O*) curve. fEPSPs were recorded in stratum radiatum using a tungsten microelectrode of 4 ± 0.8 MΩ. Data were filtered at 1-Hz high pass and 5-kHz low pass, digitized at 10 kHz using a micro3 1401 ADC (Cambridge Electronic Design, Cambridge, UK) and recorded in sweeps using signal software (CED). The initial slope (10–90%) of the FP was used as a measure for synaptic strength. LTP was induced using theta-burst stimulation (TBS) of 3 trains of 10 bursts at 5 Hz, with each burst consisting of 4 stimuli at 100 Hz and an inter train interval of 30 s.

### Statistics

Group data are presented as mean ± SD or mean ± SEM, the latter for behavioral time courses, specified in the respective figure legends. Data were analyzed with SPSS 24 and Graphpad Prism 8.0 and FlowR for IntelliCage experiments. Data were normally distributed, unless stated otherwise. Time course data or multifactorial data were submitted to two-way analysis of variance (ANOVA) using, e.g., the factors 'time' and 'genotype'. In case of significant differences, groups were mutually compared at individual time points using post hoc *t* tests according to Dunnett, i.e., versus the control group, or according to Šidák. Post hoc comparisons for between-subject factors (i.e. two genotypes or drug/vehicle) were not adjusted, if they were predefined by the experiment. In case of violations of sphericity, degrees of freedom were adjusted according to Huynh Feldt. Asterisks in figures show multiplicity-adjusted *P* values. For testing the null-hypothesis that groups were identical, we calculated the area under the behavior versus time curves using the linear trapezoidal rule and compared AUCs per unpaired two-tailed *t* tests for analyses comprising two groups or one-way ANOVA, followed by a post hoc analysis according to Šidák.

In the IntelliCage, we analyzed the number of visits and nosepokes to assess overall activity, and the licks to assess drinking behavior and the success rate. Discriminant principal component analysis (PCA) and canonical discriminant analysis (can DA) were used to reduce the complexity of behavioral parameters and assess the discrimination of the genotypes according to PCA scores or CanDisc scores. Sixteen different behavioral parameters were used as loadings. Cumulative correct visits of individual mice were plotted versus trials to assess the steepness of the learning curve. To assess the number of trials needed to achieve learning success, a probability test was used with the success criterion set to 0.35 for experiments with a random success of 0.25. Type 1 and type 2 errors were set to 0.05. The cumulative probability to achieve the respective criterion of success was plotted versus trial number to assess the proportion of learners in each group. Cosinor analysis of visiting frequencies and actograms was used to assess circadian rhythms. Mesor, acrophase and amplitude were subsequently compared between groups using unpaired, two-sided *t* tests. Social structure and cluster analyses are based on identification of leader mice and followers, i.e., which mouse entered a specific corner after the leader had left.

### RNA sequencing and analysis

Hippocampi were rapidly removed from naïve LPAR2^−/−^ and WT mice, flash frozen on dry ice, and RNA was harvested using Trizol reagent. Illumina TruSeq RNA Sample Prep Kit (TruSeq Total RNA with Ribo-Zero rRNA depletion) was used with 1 µg of total RNA for the construction of sequencing libraries. Libraries were prepared according to Illumina's instructions. Sequencing was performed an Illumina HighSeq 4000 sequencing system.

Starting with demultiplexed fastq.gz files, sequenced reads were trimmed with cutadapt(v1.18) for adapter sequences, and masked for low-complexity or low-quality sequences. The fastqc program was applied to assess sample quality, and subsequently, the alignment was done with STAR [92] using the reference genome mm10 provided from UCSC [93] as template. Results were displayed as bam files sorted by coordinates. Using FeatureCounts, a summary of bam files was created, which contained the amount of mapped reads, chromosomal locations, strand, genes and gene IDs, annotated according to the mm10 assembly. The output table was analyzed applying the Bioconductor R package Deseq2 (version 1.22.2) [94] that uses negative binomial generalized linear models to assess differential expression. Further analyses were done with ArrayStar (Lasergene) using Deseq2 normalized reads. Genes were filtered for at least three valid samples per group and normalized reads > 0.06 to exclude low expression genes. Data were log2 transformed and results displayed as scatter plots and as Volcano plots, the latter showing the log2 difference, i.e., fold change (positive for upregulated genes and negative for downregulated genes) versus the –log10 of the *t* test *P* value. The *P* value was set at 0.05 and adjusted according to Benjamini Hochberg. Hierarchical clustering was employed to assess gene expression patterns using Euclidean distance metrics. Results are displayed as heat maps with dendrograms. Key regulated genes (based on *P* value, fold change and abundance) were further analyzed for gene ontology annotation enrichments for 'cellular component', 'biological process' and 'molecular function', KEGG, Biocarta and Reactome pathways, SMART domains and SP-PIR-keywords to assess common localizations and functions of significantly regulated genes. GO analyses were done with the "term enrichment" and "functional gene clustering" tools of The Database for Annotation, Visualization and Integrated Discovery (DAVID, version 6.8) (https://david.abcc.ncifcrf.gov/home.jsp) [95]. In addition, gene set enrichment analyses (GSEA) (https://www.gsea-msigdb.org) [96] were used to further assess functional implications of up- or downregulated genes and to obtain a gene ranking and heat map of the leading edge 50 up- and downregulated genes. GSEA generates ranked gene lists based on fold difference, *P* value and abundance, which were used to create networks for the most significant genes using the web application STRING (https://string-db.org/) [97] and MIPPIE (Mouse Integrated Protein–Protein Interaction rEference; https://cbdm-01.zdv.uni-mainz.de/~galanisl/mippie/network.php) [98]. Network results were integrated into pathways using Cytoscape 3.7 [99]. The RNAseq data have been deposited as GEO dataset with the provisional accession number GSE136869.

### HPLC–MS/MS analysis of LPAR2 antagonist

LPAR2 antagonist (CAS 1017606-66-4)  concentrations were analyzed in plasma, liver and brain 1–3 h after oral administration of 50-µg antagonist soaked in 1-g cornflakes. Tissue samples were homogenized in a mixture of ethanol/water 1:3 (v/v) using a swing mill (Retsch, Haan, Germany, 25 Hz for 2.5 min) with zirconium oxide grinding balls. Samples (20 µl for plasma, 100 µl of tissue homogenates) were mixed with, respectively, 180-µl or 100-µl extraction buffer consisting in citric acid 61 mM, disodium hydrogen phosphate 77 mM, pH 4, and 20 µl of the internal standard solution containing AF38469 (200 ng/ml, Hycultec), 20-µl methanol and 600-µl ethyl acetate. Samples were then vortex-mixed for 1 min and centrifuged at 20,000 g for 5 min. For all samples, the upper organic phase was evaporated at 45 °C under a gentle stream of nitrogen and reconstituted in 50 µl of the LC/MS/MS mobile phases A:B (6:4, v/v). For the preparation of calibration standards and quality control samples, 20 µl of blank plasma (human) was processed as above and 20-µl working solution was added instead of methanol. Quality control samples of three different concentrations (low, middle, high) were run as initial and final samples of each run. Processed samples were analyzed by liquid chromatography coupled with tandem mass spectrometry (LC–MS/MS, QTrap4000, Sciex). An Agilent 1100 series binary pump (Agilent technologies, Waldbronn, Germany) equipped with a Luna C18 column (100 × 2.0 mm ID, 3 μm particle size, 100 Å pore size; Phenomenex, Aschaffenburg, Germany) was used for chromatographic separation. The column temperature was 45 °C. The HPLC mobile phases were water with 0.1% formic acid and 1-mM ammonium formate (mobile phase A), and methanol with 0.1% formic acid (mobile phase B). For separation, a gradient program was used at a flow rate of 0.35 ml/min. The initial buffer composition 60% (A)/40% (B) was linearly changed within 30 s to 30% (A)/70% (B) and then within 2 min to 0% (A)/100% (B). This composition was held for 3 min and subsequently changed within 10 s to 60% (A)/40% (B) and then held for another 2.9 min. The total run time was 8 min and the injection volume was 10 μl. The MS/MS analyses were performed using a triple quadrupole mass spectrometer API4000 (Sciex, Darmstadt, Germany) equipped with a Turbo V Ion Source operating in positive electrospray ionization mode. The MS parameters were set as follows: Ion spray voltage 5000 V, entrance potential 10 V, source temperature 400 °C, curtain gas 30 psi, collision gas 4 psi, nebulizer gas 40 psi and heating gas 60 psi. The analysis was done in Multiple Reaction Monitoring (MRM) mode with a dwell time of 100 ms. The precursor-to-product ion transition used for quantification was *m/z* 500.1 → 206.1 (collision energy 37 V, declustering potential 126 V and collision cell exit potential 16 V). Data acquisition was done using Analyst Software V 1.6.2 and quantification was performed with MultiQuant Software V 3.0.2 (both Sciex, Darmstadt, Germany). Variations in accuracy of the calibration standards were less than 15% over the whole range of calibration, except for the lower limit of quantification, where a variation in accuracy of 20% was accepted.

### Electronic supplementary material

Below is the link to the electronic supplementary material.

Supplementary file1 (PDF 1842 kb)

Supplementary file2 (DOCX 35 kb)

## Abbreviations

CUMS: Chronic unpredictable mild stress
DRL: Delayed response learning
EPM: Elevated plus maze
FA: Free adaptation
DS: Drinking session
LTP: Long-term potentiation
NP: Nosepoke
SSL: Spatial sequence learning
PAA: Place avoidance acquisition
PAEx: Place avoidance extinction
PPL: Place preference learning
PPL-Rev: Place preference learning reversal
