# Supplementary material for: Prevention of age-associated neuronal hyperexcitability with improved learning and attention upon knockout or antagonism of LPAR2
Source: Cell Mol Life Sci. 2020 May 28;78(3):1029–50. doi: 10.1007/s00018-020-03553-4 (PMC7897625; doi:10.1007/s00018-020-03553-4)
Supplement: Supplementary file 1 — Supplementary file1 (PDF 1842 kb) [file 18_2020_3553_MOESM1_ESM.pdf]

Supplementary Figures and Legends

Suppl. Fig. 1

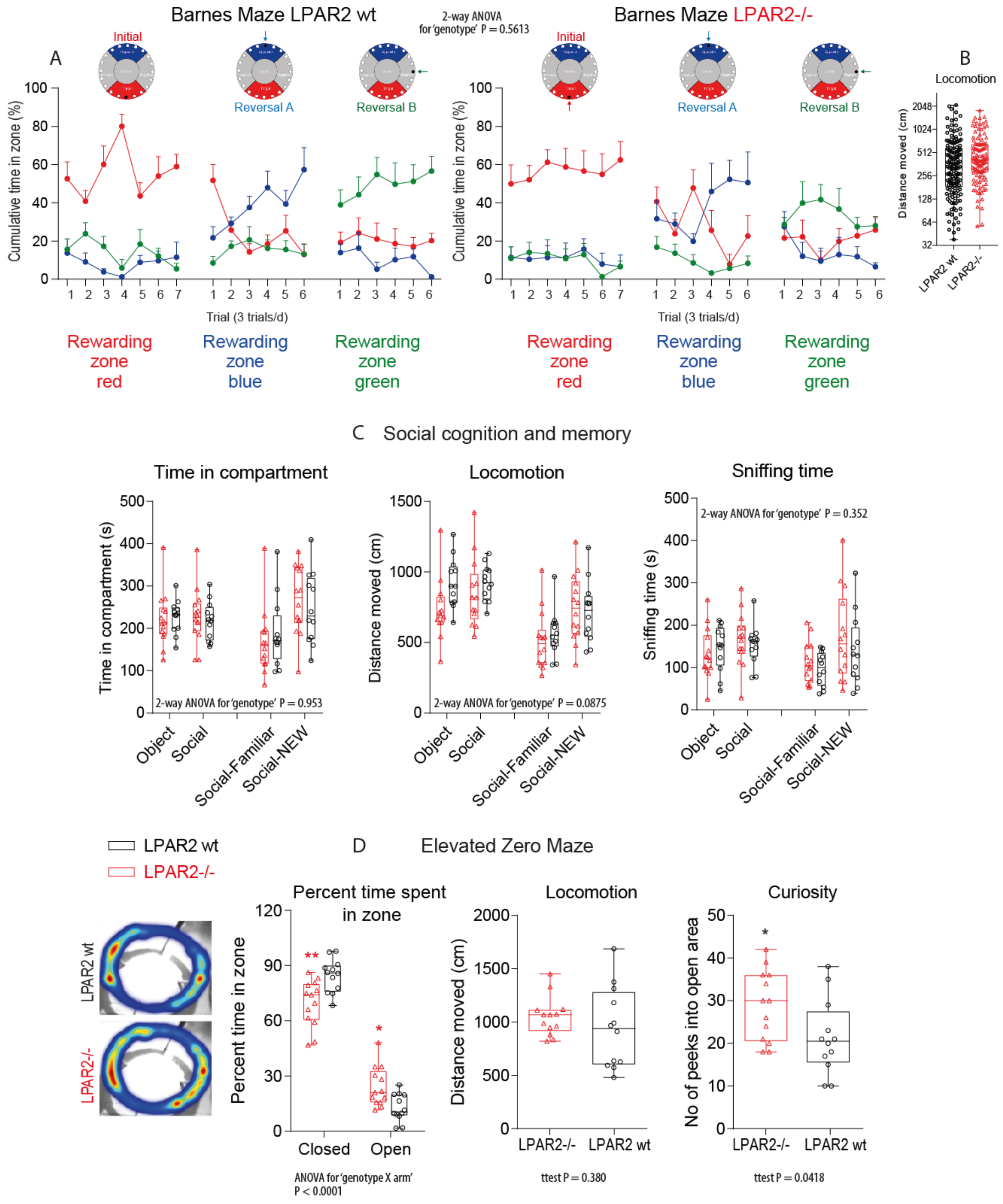

Supplementary Figure 1

Spatial and social cognition, and anxiety of wild type (LPAR2 wt) and LPAR2$^{-/-}$ mice in Barnes Maze, Social and Elevated Zero Maze tests

A: Avoidance based spatial learning and reversal learning using a standard Barnes Maze. Data show the mean ± sem of the cumulative time spent in the respective compartment as illustrated. The results are from n = 6-8 male mice per group, age at start 88-97 weeks.

B: Total distance travelled during Barnes Maze trials. All trials were pooled.

C: Social cognition and memory showing the time spent in respective compartments with object versus social partner, and familiar versus novel social partner, distances travelled per trial and sniffing times. Each test was 10 min.

D: Anxiety-like behavior in a standard Elevated Zero Maze showing the time spent in open and closed semicircles, total distance travelled during a test, which lasted for 10 min, and the number of peeks into open circles from the closed area showing the curiosity.

The data are from n = 10-14 female mice per group, age 30-40 weeks and were compared with 2-way ANOVA and subsequent t-test according to Šidák or 2-tailed unpaired t-tests in case of two groups. Asterisks indicate significant differences **P < 0.01, *P < 0.05.

Suppl. Fig 2

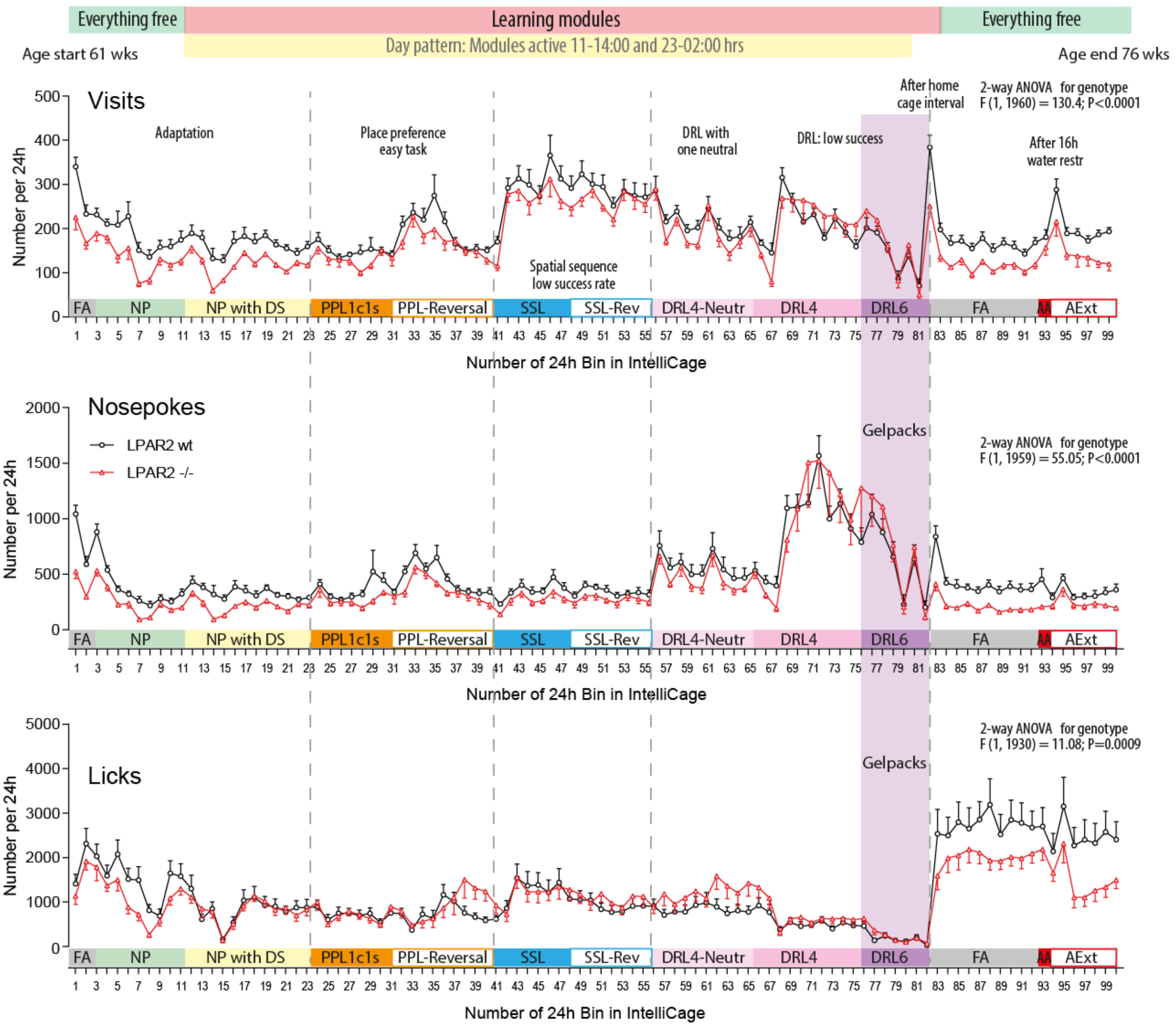

Supplementary Figure 2

Basic activity parameters over time in IntelliCage tasks

Visits, nosepokes and licks and the ratio of nosepokes per visit in 24h bins showing the general activity in the IntelliCage in female LPAR2$^{-/-}$ and LPAR2 wt mice. Mice were 60-70 weeks old at the onset of the IntelliCage experiments. The data show the mean ± sem of n = 12 mice per group, which were housed in subgroups of 6/6 of each genotype per IntelliCage throughout the experiments, with few home cage interruptions required by specific tasks or for cage cleaning reasons.

The time schedule of the different tasks is illustrated below the licks. The adaptation started with "**free adaptation"** (**FA**), during which all doors were open and water freely available. This was followed by "**nosepoke adaptation"** (**NP**), during which all doors were closed but could be opened with a NP at the door for 5 s. The NP settings were maintained during the following experiments.

During **"drinking session"** (**DS**) mice had to learn to get water only during 2x3 hours per day, 11:00-14:00 and 23:00-02:00. This day pattern was maintained through the following tasks up to "Delayed Response

Learning". Outside of the drinking periods, doors remained closed and learning modules were switched to default modules ensuring that all doors remained closed and LEDs were off.

During **"place preference learning"**, **PPL1c1s, and PPL-Reversal** mice had to learn to prefer one corner and one side of this corner, in which doors opened on a correct nosepoke on the correct side and gave access to tap water as reward. Each 4 mice each were assigned to one corner. In the reversal period (PPL-rev) the awarding corner and door were switched to the opposite corner of the cage and opposite side within the corner.

In the **Spatial Sequence Learning** protocol (SSL), mice had to learn that the next awarding corner was adjacent to the presently rewarding corner in clockwise (SSL) or anti-clockwise (SSL-rev) direction. The experiment assesses spatial learning capabilities. It is a difficult task and forces the animals to increase their visiting frequencies. The corner switch was executed only after successful drinking to ensure that the mice got the reward. If they did not drink in the correct corner, this corner remained "correct" until mice drank in this corner. The entry of a correct corner was announced by green LED, which was switched off after drinking or leaving the corner.

In the **Delayed Response Learning task**, mice had to learn to make repetitive nosepokes at the same door but at a low rate with an increasing delay between the first and second nosepoke of 4s or 6s. In the initial training (DRL4s-1neutral) one corner was defined as neutral, in which a single nosepoke opened the door but only with a probability of 50%. Subsequently, the neutral corner was closed in DRL4s and DRL6s. The DRL task is difficult and mice of both genotypes increased the visiting frequencies. Success was mostly random. During DRL6s rescue packs were provided overnight because lickings of some animals dropped below a critical amount.

After the DRL mice returned to their home cage. When they returned they were readapted with the "Free adaptation" protocol to assess unrestricted behavior and circadian rhythms.

During '**Airpuff**' (**place avoidance acquisition**, **PAA**) mice had to learn to avoid one corner, in which a nosepoke elicited an airpuff and doors remained closed. The acquisition was for 24 h, followed by 24 h in the home cage. On returning to the IntelliCage for evaluation of the maintenance of corner-avoidance (**Place avoidance extinction**, **PAEx**) mice were allowed to use all corners freely, using an NP protocol without restrictions.

Suppl. Fig 3

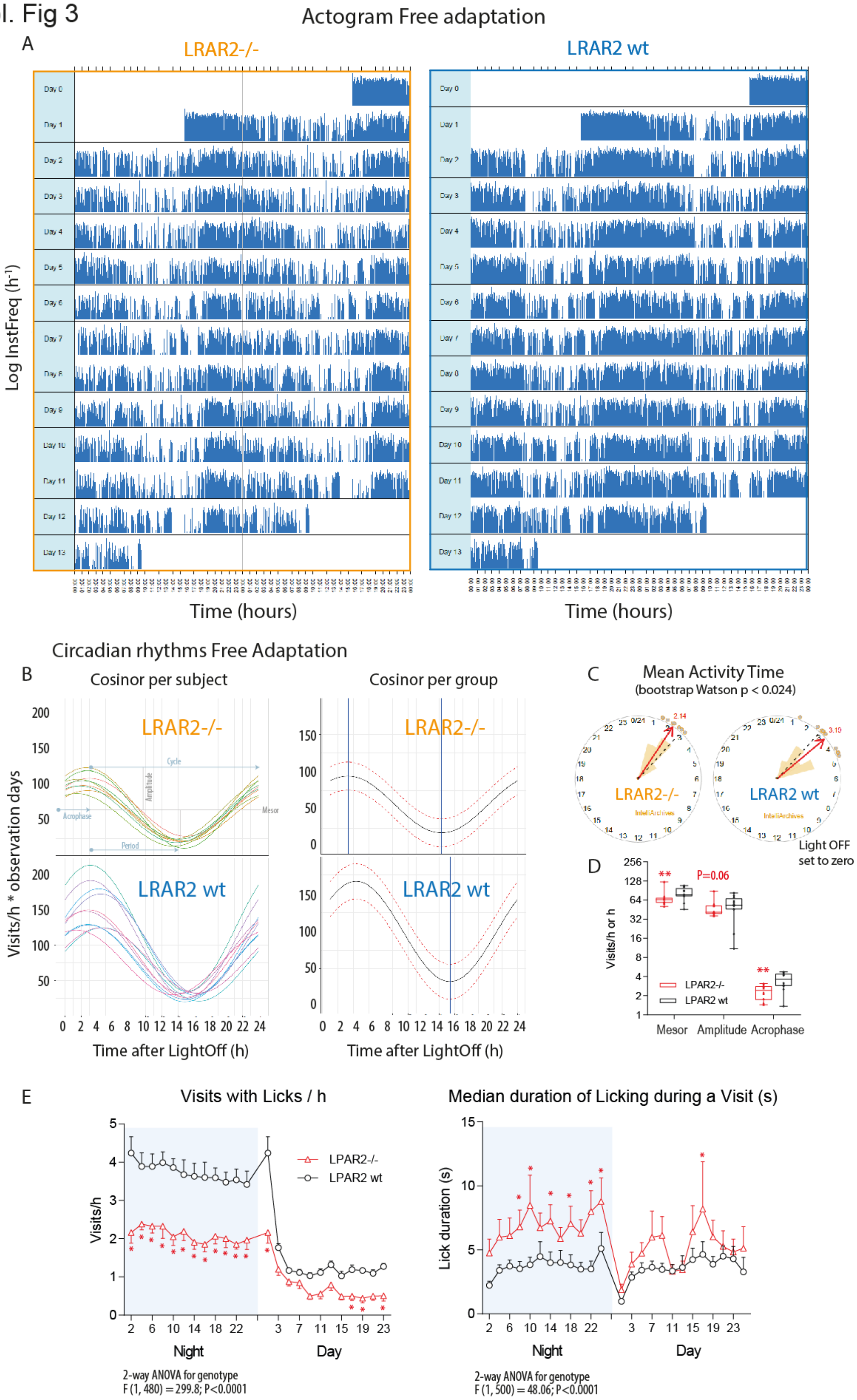

Actogram Free adaptation
A
LRAR2-/-
LRAR2 wt
Log InstFreq (h-1)
Time (hours)
Time (hours)
Circadian rhythms Free Adaptation
B
Cosinor per subject
Cosinor per group
LRAR2-/-
LRAR2 wt
Visits/h * observation days
Time after LightOff (h)
Time after LightOff (h)
C
Mean Activity Time
(bootstrap Watson p < 0.024)
LRAR2-/-
LRAR2 wt
Light OFF
set to zero
D
Visits/h or h
LPAR2-/-
LPAR2 wt
P=0.06
Mesor
Amplitude
Acrophase
E
Visits with Licks / h
Median duration of Licking during a Visit (s)
Visits/h
Lick duration (s)
LPAR2-/-
LPAR2 wt
Night
Day
Night
Day
2-way ANOVA for genotype
F (1, 480) = 299.8; P<0.0001
2-way ANOVA for genotype
F (1, 500) = 48.06; P<0.0001

Supplementary Figure 3

Actograms and cosinor analysis of circadian rhythms

Circadian analysis was done for Free Adaptation in the IntelliCage in LPAR2$^{-/-}$ and LPAR2 wt female mice, n = 12 per group. Mice were housed in groups of 6/6 of each genotype per IntelliCage, and were 65-85 weeks old.

A: Actograms reveal a higher exploratory activity in LPAR2 wt animals. Both genotypes show clear circadian rhythms.

B-D: Cosinor analysis for animals and groups shows the higher visiting frequency and higher fluctuations of night-to-day activity in LPAR2 wt animals resulting in higher mesor and amplitude. The acrophase of LPAR2$^{-/-}$ mice preceded the wt acrophase for about one hour. During experiments with restricted drinking/learning times, circadian rhythms were synchronized (acrophase 4-6 h after light off, i.e. during the night drinking period).

E: Circadian phased analysis of Visits/h and 'median duration of lickings during a visit' during Free Adaptation in the IntelliCage. LPAR2$^{-/-}$ make substantially fewer visits throughout this experiment but compensate the lower visiting frequency with longer licking times during their visits. The data show the mean ± sem of 12 mice per group.

Suppl. Fig 4

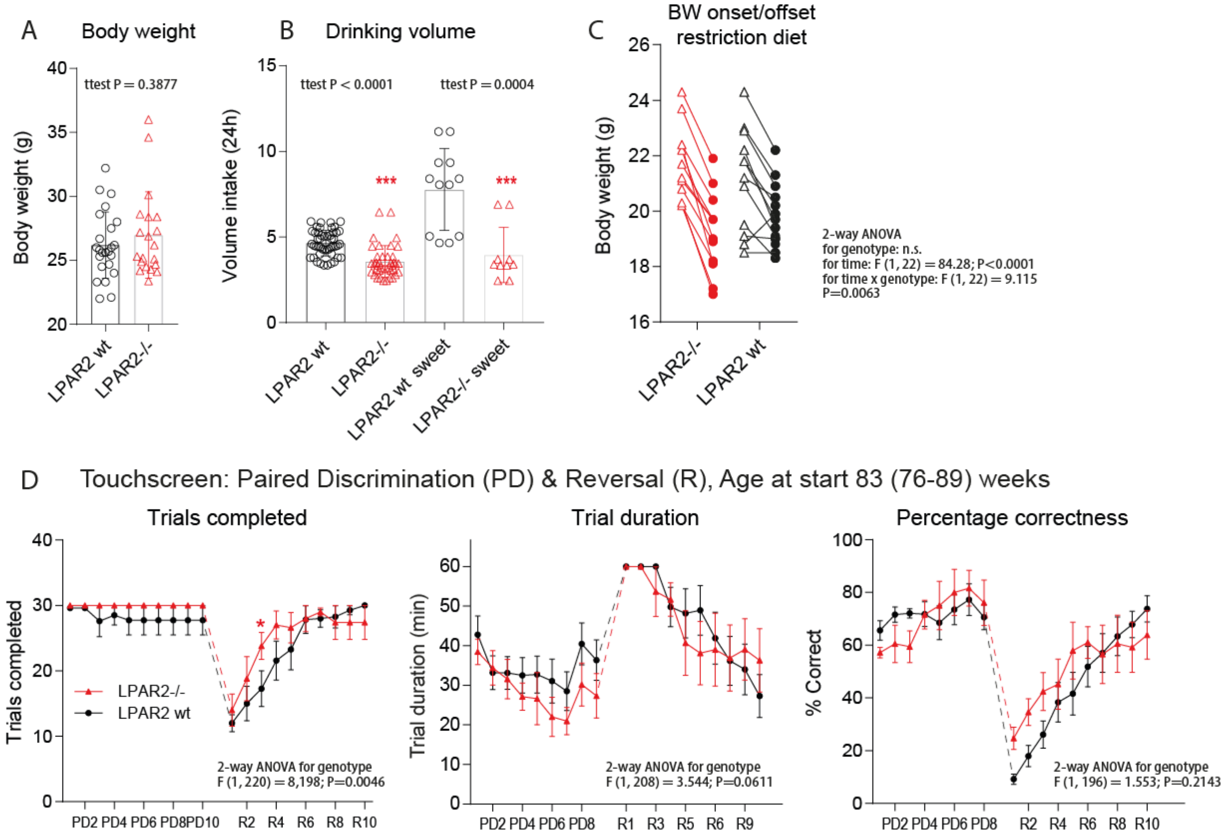

Supplementary Figure 4

Body weight, sucrose preference and perseverations

A: Baseline bodyweights of 45-65 weeks old female LPAR2$^{-/-}$ and LPAR2 wt mice (n = 10, 12). Each mouse is represented twice, at onset and end of the experiment.

B: Drinking volume per day with free drinking and 2-choice bottles with water or sucrose-water (2%)

C: Body weights at onset and end of a 10% reduction diet during touchscreen experiments. LPAR2$^{-/-}$ lost more weight during the diet than controls (2-way ANOVA 'genotype' x 'time').

D: Time course of the accuracy of touchscreen touches and number of perseverations during 5CSRT task before and during CUMS. Stressors during CUMS are described in Suppl. Table 4. Data were compared with 2-way ANOVA for 'genotype' x 'trial'.

Suppl. Fig 5

GSEA scores of leading edge 50 up and 50 down regulated

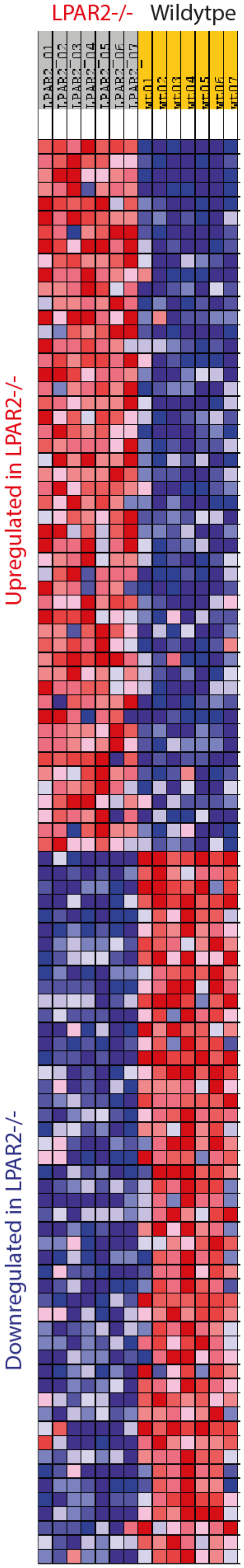

PLVAP	plasmalemma vesicle associated protein
LPL	lipoprotein lipase
FGF10	fibroblast growth factor 10
MAMDC2	MAM domain containing 2
HDHD3	haloacid dehalogenase-like hydrolase domain containing 3
OCEL1	occludin/ELL domain containing 1
TLR1	toll-like receptor 1
DBP	D site of albumin promoter (albumin D-box) binding protein
TWIST1	twist homolog 1 (acrocephalosyndactyly 3; Saethre-Chotzen syndrome) (Drosophila)
CAPS2	calcyphosine 2
FMO2	flavin containing monooxygenase 2 (non-functional)
PRR15	proline rich 15
SLAMF8	SLAM family member 8
COL22A1	collagen, type XXII, alpha 1
ALPK3	alpha-kinase 3
IFI30	interferon, gamma-inducible protein 30
NANOS3	nanos homolog 3 (Drosophila)
TNFRSF13C	tumor necrosis factor receptor superfamily, member 13C
PLA2G4E	phospholipase A2, group IVE
CREB3L4	cAMP responsive element binding protein 3-like 4
MARVELD2	MARVEL domain containing 2
PBX4	pre-B-cell leukemia transcription factor 4
TRH	thyrotropin-releasing hormone
CCDC67	coiled-coil domain containing 67
CLSPN	claspin homolog (Xenopus laevis)
EIF2AK2	eukaryotic translation initiation factor 2-alpha kinase 2
UPP1	uridine phosphorylase 1
PLAU	plasminogen activator, urokinase
IL5	interleukin 5 (colony-stimulating factor, eosinophil)
ACR	acrosin
DMRTB1	DMRT-like family B with proline-rich C-terminal, 1
TDRD6	tudor domain containing 6
FSTL3	follistatin-like 3 (secreted glycoprotein)
TBC1D10C	TBC1 domain family, member 10C
SFRP1	secreted frizzled-related protein 1
ITK	IL2-inducible T-cell kinase
NANOS2	nanos homolog 2 (Drosophila)
IL12RB1	interleukin 12 receptor, beta 1
FILIP1	filamin A interacting protein 1
HDDC3	HD domain containing 3
DNAH8	dynein, axonemal, heavy chain 8
LZTS1	leucine zipper, putative tumor suppressor 1
PGM5	phosphoglucomutase 5
ALAD	aminolevulinate, delta-, dehydratase
SLC16A12	solute carrier family 16, member 12 (monocarboxylic acid transporter 12)
COL8A2	collagen, type VIII, alpha 2
TUSC1	tumor suppressor candidate 1
TEX11	testis expressed sequence 11
COL8A1	collagen, type VIII, alpha 1
LST1	leukocyte specific transcript 1
SCOC	short coiled-coil protein
FGL1	fibrinogen-like 1
SLC5A5	solute carrier family 5 (sodium iodide symporter), member 5
FUT10	fucosyltransferase 10 (alpha (1,3) fucosyltransferase)
FOSB	FBJ murine osteosarcoma viral oncogene homolog B
CDKN1A	cyclin-dependent kinase inhibitor 1A (p21, Cip1)
HMGB2	high-mobility group box 2
DUSP5	dual specificity phosphatase 5
CRLF1	cytokine receptor-like factor 1
SERINC2	serine incorporator 2
PTTG1	pituitary tumor-transforming 1
NPAS4	neuronal PAS domain protein 4
HPGD	hydroxyprostaglandin dehydrogenase 15-(NAD)
FOS	v-fos FBJ murine osteosarcoma viral oncogene homolog
CHCHD2	coiled-coil-helix-coiled-coil-helix domain containing 2
ABCB10	ATP-binding cassette, sub-family B (MDR/TAP), member 10
IL27RA	interleukin 27 receptor, alpha
UBC	ubiquitin C
HSPB8	heat shock 22kDa protein 8
ARC	activity-regulated cytoskeleton-associated protein
PLA2G2F	phospholipase A2, group IIF
NPTX2	neuronal pentraxin II
GPR17	G protein-coupled receptor 17
DOK3	docking protein 3
EGR2	early growth response 2 (Krox-20 homolog, Drosophila)
TDGF1	teratocarcinoma-derived growth factor 1
NR4A2	nuclear receptor subfamily 4, group A, member 2
EPS8L1	EPS8-like 1
HIST1H2BE	histone cluster 1, H2be
HIST1H1E	histone cluster 1, H1e
HSPA5	heat shock 70kDa protein 5 (glucose-regulated protein, 78kDa)
ACP1	acid phosphatase 1, soluble
FAM89A	family with sequence similarity 89, member A
TMEM100	transmembrane protein 100
FOSL2	FOS-like antigen 2
THBS4	thrombospondin 4
B3GNT3	UDP-GlcNAc:betaGal beta-1,3-N-acetylglucosaminyltransferase 3
ANTXR2	anthrax toxin receptor 2
RAB3B	RAB3B, member RAS oncogene family
IRX1	iroquois homeobox protein 1
ARL2BP	ADP-ribosylation factor-like 2 binding protein
BUB1B	BUB1 budding uninhibited by benzimidazoles 1 homolog beta (yeast)
ELOVL1	elongation of very long chain fatty acids (FEN1/Elo2, SUR4/Elo3, yeast)-like 1
PDIA6	protein disulfide isomerase family A, member 6
SDF2L1	stromal cell-derived factor 2-like 1
PHKG1	phosphorylase kinase, gamma 1 (muscle)
RECQL4	RecQ protein-like 4
SGOL1	shugoshin-like 1 (S. pombe)
TBX1	T-box 1
BFSP2	beaded filament structural protein 2, phakinin

Supplementary Figure 5

Heat maps of GSEA ranked genes

Leading edge upregulated and downregulated 50 candidate genes in LPAR2$^{-/-}$ versus wildtype mice. The color scale ranges from red (upregulated) to blue (downregulated). The ranking is based on P-value, q-value and fold change.

Suppl. Figure 6

A

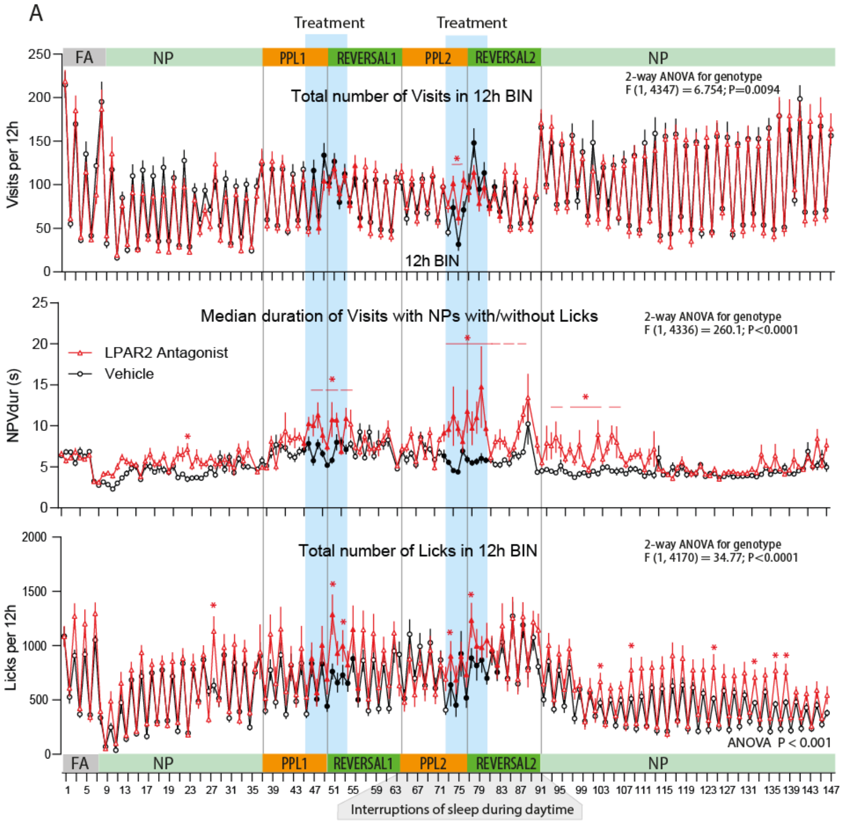

Blue shaded areas:
Period of antagonist/vehicle adminsitration
FA: Free Adaptation
NP: Nosepoke Adaptation
PPL: Place Preference Learning (1corner2sides)
one correct corner, both sides
Reversal: Switch of rewarding corners to
opposite side

B

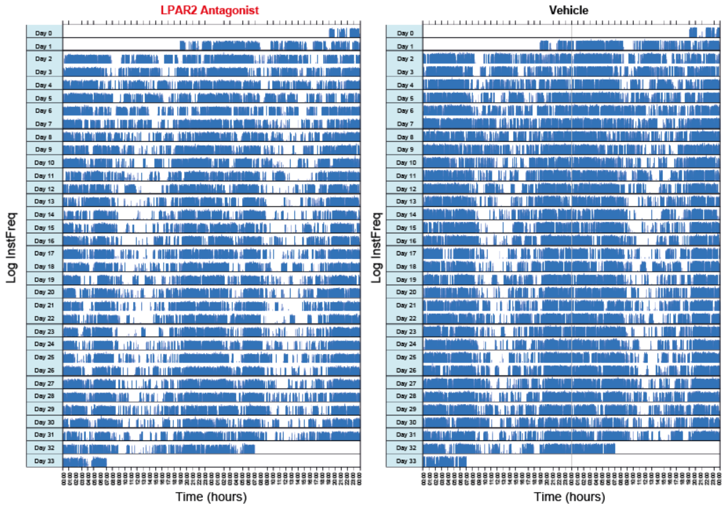

Actograms following
a period of unpredictable
sleep/circadian rhythm disruptions
LPAR2-antagonist treated mice
resume circadian
rhythms faster than
vehicle treated mice

Supplementary Figure 6

IntelliCage behavior in mice treated with LPAR2 inhibitor or vehicle

Female mice were treated with 50 µg LPAR2-inhibitor in 2 grams cornflakes (n=13) or vehicle cornflakes (n=12) once daily in the morning. During the second Place Preference Learning period (PPL2 & REVERSAL2), mice were exposed to daytime sleep disruptions causing mild stress. Mice were 74-75 weeks old at the onset of the experiment.

A: Time courses of key behavioral parameters. Mice were adapted with free adaptation (FA) and nosepoke adaptation (NP) and put on a mild restriction diet (3 grams of pellets per mouse and day) to increase the appetite for the medication-cornflakes. Place preference learning (PPL1) was started and cornflakes were introduced as morning meal. The treatment period encompassed the last two days of PPL1 (or PPL2) up to the third day after Reversal of PPL1 (or Reversal PPL2) (filled symbols, blue shaded areas). Reversal refers to the switch of the rewarding corner to the opposite site. The first learning and reversal (PPL1 & Reversal) was without any disruptions, and the second PPL2 and Reversal was done with daily disruptions of daytime sleep in random order explained in Suppl. Table 5.

The data are means ± sem. Differences between groups were assessed with 2-way ANOVA. Asterisks point to significant time points or periods for the between subject factor "group" (P not adjusted for multiple time point assessment). Dashed lines indicate that not all consecutive individual time points were significant. The tasks and daytime sleep disruptions during PPL2/REVERSAL2 are explained in Suppl. Table 2 and Suppl. Table 5, respectively.

B: Actograms show the activity in LPAR2-antagonist and vehicle treated mice in the post-stress/post treatment period with free access to all corners. LPAR2-antagonist treated mice regained circadian rhythms faster than vehicle treated mice, and they showed lower daytime activity suggesting fewer sleep interruptions.
