# Supplementary material for: Prevention of age-associated neuronal hyperexcitability with improved learning and attention upon knockout or antagonism of LPAR2
Source: Cell Mol Life Sci. 2020 May 28;78(3):1029–50. doi: 10.1007/s00018-020-03553-4 (PMC7897625; doi:10.1007/s00018-020-03553-4)
Supplement: Supplementary file 2 — Supplementary file2 (DOCX 35 kb) [file 18_2020_3553_MOESM2_ESM.docx]

**Supplementary Tables**

*Suppl. Table 1:*

#### Behavioral groups and experiment schedules

| **Cohort & sequence** | **Genotype** | **Experiment** | **Sample size (n)** | | **Age at start of experiment (wks)** | | | **Mean age end of experiment** |
| --- | --- | --- | --- | --- | --- | --- | --- | --- |
|  |  |  | male | female | **Mean** | Min | Max |  |
| A-1 | LPAR2-/- | Touchscreen - 5CSRT | 8 |  | **75** | 66 | 80 | **85** |
| A-1 | LPAR2+/+ | Touchscreen - 5CSRT | 8 |  | **70** | 65 | 75 | **81** |
| A-2 | LPAR2-/- | Touchscreen - PD | 7 |  | **85** | 77 | 89 | **92** |
| A-2 | LPAR2+/+ | Touchscreen - PD | 8 |  | **81** | 76 | 83 | **88** |
| A-3 | LPAR2-/- | Barnes Maze | 6 |  | **94** | 88 | 97 | **96** |
| A-3 | LPAR2+/+ | Barnes Maze | 8 |  | **90** | 87 | 92 | **92** |
| B-1 | LPAR2-/- | Social Cognition & Memory |  | 14 | **36** | 27 | 40 | **36** |
| B-1 | LPAR2+/+ | Social Cognition & Memory |  | 12 | **24** | 21 | 27 | **24** |
| B-2 | LPAR2-/- | Zero Maze |  | 13 | **36** | 28 | 41 | **36** |
| B-2 | LPAR2+/+ | Zero Maze |  | 12 | **24** | 22 | 28 | **24** |
| B-3 | LPAR2-/- | Touchscreen - 5CSRT |  | 12 | **37** | 28 | 41 | **54** |
| B-3 | LPAR2+/+ | Touchscreen - 5CSRT |  | 12 | **25** | 22 | 28 | **42** |
| B-4 | LPAR2-/- | Intellicage |  | 11 | **67** | 58 | 71 | **82** |
| B-4 | LPAR2+/+ | Intellicage |  | 12 | **55** | 51 | 58 | **70** |
| C-1 | Antagonist | Intellicage |  | 13 | **74** | 71 | 78 | **85** |
| C-1 | Vehicle | Intellicage |  | 12 | **75** | 71 | 78 | **86** |

The age is in weeks.

5CSRT: 5 Choice Serial Reaction Time Task; CUMS: Chronic unpredictable mild stress

*Suppl. Table 2*

#### Summary of the protocols and behavioral tasks in the IntelliCage

| Task name | Task description | Duration | Drinking time | Cognitive structures |
| --- | --- | --- | --- | --- |
| Free adaptation (FA) | Habituation to the system with free access to every corner, with all doors open, and water and food ad libitum. | 3-4 days | No restriction |  |
| Nosepoke adaptation (NP) | The first nosepoke of a visit opened the door for 5 s. To drink more, the animals had to leave the corner and start a new visit. | 9 days up to 6 weeks | No restriction |  |
| Nosepoke drinking session (DS) | To increase the learning drive, mice were adjusted to defined drinking sessions, in which the “nosepoke” task was active. The start of the drinking time was announced with green LED lights for 2 min. | 12 days | Restricted to 11-2 a.m. and 11-2 p.m. | Hippocampus required for adaptation to temporal circadian patterns [1] |
| Place preference learning (PPL) with drinking session | Mice were allowed to drink in only one corner. To cause social competition, all mice were assigned to the same corner. During day-time DS the left side of the corner was correct with a green LED; in the night DS the right side of the corner was correct with red LED. Only the first correct nosepoke of a visit opened the door. | 8 days | Restricted to 11-2 a.m. and 11-2 p.m. | Hippocampal dysfunction spares simple spatial learning tasks but impairs the ability to cope with conflicting tasks with inherent spatial, temporal or emotional cues [1] |
| Place preference REVERSAL (PPLrev) | Protocol as in “place preference learning” but with the opposite corner / sides being correct. | 10 days | Restricted to 11-2 a.m. and 11-2 p.m. | Flexible spatial reversal learning requires the dorsal and ventral hippocampus and their functional interactions with the prefrontal cortex [2, 3] |
| Spatial sequence learning (SSL) | Mice had to learn that the next rewarding corner was in clockwise rotation compared with actual rewarding corner. A correct visit was indicated with green LED | 11 days | Restricted to 11-2 a.m. and 11-2 p.m. | Spatial sequence learning critically depends on the hippocampus and its connectivity to the retrosplenial cortex [4] |
| Spatial sequence REVERSAL (SSLrev) | Same protocol as in “Spatial sequence learning” but with anti-clockwise rotation. | 15 days | Restricted to 11-2 a.m. and 11-2 p.m. |  |
| Delayed response learning (DRL) | Mice had to poke a second time on the same door with a defined delay in order to get a water reward.  The training consisted in 3 periods: 4 s delay with a “neutral” corner; 4 s delay; 6 s delay. In the “neutral” corner, one NP opened the door in 50% of the visits. | 27 days | Restricted to 11-2 a.m. and 11-2 p.m. | Control of waiting impulsivity requires functions of the hippocampus and medial PFC [5, 6] |
| Place avoidance acquisition (PAA) | Mice had to avoid a specific corner. NP in this corner was punished with an air-puff and a red LED was switched on upon visit to this corner. Avoidance acquisition was for 24h, followed by aone-day home cage interval. | 1 day | No restriction | The PAA/PAex is sensitive to genetic differences and hippocampal lesions [7] |
| Place avoidance extinction (PAex) | During avoidance extinction, water was available in each corner on NP without any punishment. Only the red LED still announced the previously punished corner. | 6 days | No restriction |  |
| Place preference learning with LPAR2 inhibitor | Mice were allowed to drink in only one corner, on both sides.  The experiment was done without (7d) and with disruptions of circadian rhythms (7d) | 2 x 7 days | No restriction | PPL with additional circadian stresses and repeated Reversals depends on the hippocampus, PFC, amygdala and Nc. accumbens |
| Place preference learning and REVERSAL with LPAR2 inhibitor | Protocol as in “place preference learning with LPAR2 antagonist” but with the opposite corner being correct. | 2 x 7 days | No restriction |  |

*Suppl. Table 3*

#### Abbreviations of behavioral parameters of IntelliCage experiments

| Visits | Visits / h |
| --- | --- |
| NPvisits | Visits with Nosepoke without Licks / h |
| Lvisits | Visits with Licks / h |
| SVisits | Visits without Licks and without Nosepokes / h |
| NPVdur | Median duration of Visits with NP w/out Lick (s) |
| Nosepokes | Mean number of Nosepokes during Visits with NP w/out Licks |
| NPduration | Median duration of such Nosepokes during a Visit (s) |
| Licks | Median number of Licks per Visit |
| Lduration | Median duration of Licking during a Visit (s) |
| Lcontact | Median bottle cap contact time during a Visit (s) |
| Nocturnal | Log(Visit frequency during dark phase / Visit frequency during light phase) |
| Repetitive | Repetitiveness, log(sum of observed returns to same corner / sum of expected such switches) |
| Regularity | Sqrt (sum of sq non-diag. transition matrix residuals / sum of non-diag. transition matrix observed values) |
| IVI | Intervisit intervals (s) i.e. time from end of visit to start of next corner visit |
| IVIrepdens | Intervisit intervals (s) for repeated use of the same corner |
| InstantFreq | Instantaneous frequency, reciprocal of the time from start of one visit to start of next visit |
| Unevenness | Describes the relative use of corners, ranges from 0-1 (0=equal use of 4 corners, 1=exclusive use of 1 corner) |
| Sidedness | Ratio of visits with first left versus first right NP of visits with NPs |
| Mesor | **M**idline **e**stimating **s**tatistic **o**f **r**hythm, a rhythm adjusted mean in cosinor analysis |
| Amplitude | Difference between Mesor and Peak activity |
| Acrophase | Time to maximum activity after Light Off (Light off set to 0) |
| Period | Duration of one cycle |

*Suppl. Table 4*

#### Daily stressors in the sleep interruption (CUMS) protocol

| Stressor | Description |
| --- | --- |
| Food every hour after overnight fasting | Few food pellets were provided every hour starting at 10 am. Alternatively, few cornflakes were provided. Cornflakes were used during cornflakes habituation, and food pellets in Reversal phases. |
| Repeated bedding change | Bedding and housing were exchanged repeatedly throughout the day |
| 36h darkness | The light was off for 36h |
| 2h Light ON/OFF cycles | The light was turned on and off every 2h for 24h and red houses were removed intermittently. |

Stressors were applied in pseudorandom order over a period of 10 days.

*Suppl. Table 5*

#### Touchscreen training and tasks: 5CSRT (5-choice serial reaction time) and PD (pairwise discrimination)

| # | Touchscreen Experiment | Touchscreen task name | Description | sessions | Criterion for success |
| --- | --- | --- | --- | --- | --- |
| 0 | 5CSRT and PD | Motivation diet | Food pellets are reduced to 1-2.5 g per mouse/day to reduce and keep bodyweight at 90% of baseline. In paralle mice are habituated to sweetened condensed milk (1:4 in tap water) | 3-7 days | 10% reduction diet (for adult, not growing mice) |
| 1 | 5CSRT and PD | Cage Habituation | Mice are set in TS cage for 40 min and receive 150 µl sweetened condensed milk | 2 | Mice must drink the offered sweetened condensed milk completely to reach the next phase |
| 2 | 5CSRT and PD | Habituation 2v2 | Mice start with 150 µl sweetened condensed milk (prime), then mice get every 10 ses 8 µl sweetened condensed milk along with a signal tone, so that mice associate the signal tone with the reward | 2 | Mice must complete 30 trials during the session (60 min) for 2 consecutive days |
| 4 | 5CSRT and PD | Must Touch | The screen displays an image of five rectangles, and mice have to touch it (nosepoke) in order to receive reward coupled to the signaling tone | 2 | Mice must complete 30 correct trials (touching the image on the screen) during the session (60 min) for 2 consecutive days |
| 5 | 5CSRT and PD | Must Intiate Touch | Mice must intiate the image display by putting the head into the food tray, and then make a nosepoke on the screen to receive their reward. | 4 | Mice must complete 30 correct trials (touching the picture on the screen) and initiate the next image display during the session (60 min) for 2 consecutive days |
| 6 | 5CSRT and PD | Punish Incorrrect | As Must Initiate Touch, but a nosepoke on the screen outside the image is considered as a failed trial. In consequence the house light goes on along with a time out of 5 sec and 5 sec ITI (inter trial interval), and the picture disappears. Mice have to initiate the next display of the picture by putting their head into the food tray. For **5CSRT:** A white square appears in 1 out of 5 possible positions. For **Pairwise Discrimination** (PD): an image (a random picture from a selection of 40 images) is presented in one of the two positions | 6-9 | Mice must complete 30 trials during one session (60 min) with a correctness of >60-75% (depends on age) for 3 consecutive days. A correct trial is a nosepoke on the screen in the correct position. |
| 7 | 5CSRT: 5-Choice Serial Reaction Time Task | 5CSRT Basic v3 | Same as in Punish Incorrect, but the display time of the picture (Stimulus duration) is restricted to 32, 16, 8, 4 or 2 s. If the mouse does not react to the stimulus (omission) or performs an incorrect touch, there will be a punishment (timeout) as in Punish Incorrect. | 3 per stimulus time | Mice must complete 40-60 trials during one session (60 min) with a correctness of >60-75% depending on age and an omission rate <20% for 2 consecutive days. If >75% of mice fail, the experiment is finished. |
| 7 | PD: Discrimination of pairwise presented easy pictograms such as flower and airoplane | Pairwise Discrimination v3 (Initial) | Mice learn to discriminate between two novel images. One is randomly designated as "correct". The images are presented on right or left side pseudo-randomized. The Intertrial INterval (ITI) is 15 sec. Correct responses are rewarded. Each incorrect response is followed by a correction trial, in which the images is presented as in the previous trial, until a correct response is made. | 6 | Mice must complete 30 trials during one session (60 min) with a correctness of >60-75% depending on age for 3 consecutive days |
| 8 | PD REVERSAL | Pairwise Discrimination v3 (Reversal) | Settings like in PD Intial, but the designation of correct and incorrect images is switched, i.e. the previously rewarded picture is now incorrect and vice versa | 8 | Mice must complete 30 trials during one session (60 min) with correctness of >60-75 % depending on age for 3 consecutive days |

*Suppl. Table 6*

#### Ages of mice used for brain slice preparation

| Experiment | Age range (weeks) | WT age mean ± SD | LPAR2^-/-^ age mean ± SD |
| --- | --- | --- | --- |
| I/O Young | 13-16 | 14.6 ± 1.1 | 13.2 ± 0.4 |
| I/O Old | 49-81 | 69.0 ± 12.9 | 55 ± 4.6 |
| LTP Young | 10-14 | 11 ± 1 | 13.2 ± 0.4 |
| LTP Old | 35-81 | 64.0 ± 25 | 36 ± 0.6 |

Slices were prepared from 3-5 mice per group.

**References**

1. Voikar V, Krackow S, Lipp HP, et al. (2018) Automated dissection of permanent effects of hippocampal or prefrontal lesions on performance at spatial, working memory and circadian timing tasks of C57BL/6 mice in IntelliCage. Behav Brain Res 352:8-22

2. Avigan PD, Cammack K, Shapiro ML (2020) Flexible spatial learning requires both the dorsal and ventral hippocampus and their functional interactions with the prefrontal cortex. Hippocampus;10.1002/hipo.23198

3. Vila-Ballo A, Mas-Herrero E, Ripolles P, et al. (2017) Unraveling the Role of the Hippocampus in Reversal Learning. J Neurosci 37:6686-97

4. Mao D, Neumann AR, Sun J, et al. (2018) Hippocampus-dependent emergence of spatial sequence coding in retrosplenial cortex. Proc Natl Acad Sci U S A 115:8015-8

5. Cho YH, Jeantet Y (2010) Differential involvement of prefrontal cortex, striatum, and hippocampus in DRL performance in mice. Neurobiol Learn Mem 93:85-91

6. Jackson PA, Kesner RP, Amann K (1998) Memory for duration: role of hippocampus and medial prefrontal cortex. Neurobiol Learn Mem 70:328-48

7. Voikar V, Colacicco G, Gruber O, et al. (2010) Conditioned response suppression in the IntelliCage: assessment of mouse strain differences and effects of hippocampal and striatal lesions on acquisition and retention of memory. Behav Brain Res 213:304-12
